# Central and peripheral circadian clocks and their role in Alzheimer's disease

**DOI:** 10.1242/dmm.030627

**Published:** 2017-10-01

**Authors:** Ruchi Chauhan, Ko-Fan Chen, Brianne A. Kent, Damian C. Crowther

**Affiliations:** 1 Department of Genetics, University of Cambridge, Downing Street, Cambridge, CB2 3EH, UK; 2 Institute of Neurology, UCL, London, WC1N 3BG, UK; 3 Djavad Mowafaghian Centre for Brain Health, University of British Columbia, 2215 Wesbrook Mall, Vancouver, V6T 1Z3, Canada; 4 Neuroscience, Innovative Medicines and Early Development, AstraZeneca, Granta Park, Cambridge, CB21 6GH, UK

**Keywords:** Circadian biology, Clearance, Protein aggregation, Proteostasis, Sleep dysfunction

## Abstract

Molecular and cellular oscillations constitute an internal clock that tracks the time of day and permits organisms to optimize their behaviour and metabolism to suit the daily demands they face. The workings of this internal clock become impaired with age. In this review, we discuss whether such age-related impairments in the circadian clock interact with age-related neurodegenerative disorders, such as Alzheimer's disease. Findings from mouse and fly models of Alzheimer's disease have accelerated our understanding of the interaction between neurodegeneration and circadian biology. These models show that neurodegeneration likely impairs circadian rhythms either by damaging the central clock or by blocking its communication with other brain areas and with peripheral tissues. The consequent sleep and metabolic deficits could enhance the susceptibility of the brain to further degenerative processes. Thus, circadian dysfunction might be both a cause and an effect of neurodegeneration. We also discuss the primary role of light in the entrainment of the central clock and describe important, alternative time signals, such as food, that play a role in entraining central and peripheral circadian clocks. Finally, we propose how these recent insights could inform efforts to develop novel therapeutic approaches to re-entrain arrhythmic individuals with neurodegenerative disease.

## Abstract (precis)

**Summary:** This Review summarizes how studies in *Drosophila* and mouse models accelerate our understanding of the links between circadian rhythm dysfunctions and age-related neurodegenerative disorders, especially Alzheimer's disease.

## Introduction

The circadian clock is a complex biological machine that allows organisms, from fruit flies to humans, to predict and prepare for the challenges of everyday life. According to each organism's ecological niche, activities such as sleeping, eating, mating and predation-avoidance are optimally performed either during the day or night (Hut et al., 2012). This ability to track the hours of the day must, therefore, be of general benefit, as evidenced by the remarkable conservation of the molecular components of the circadian clock across many species (for exceptions see Bloch et al., 2013). Given the importance of circadian biology in regulating organismal health, it is of no surprise that the breakdown of daily circadian rhythms (see Glossary, Box 1) is a risk factor for a range of diseases, including metabolic syndrome, vascular disease and cancer (Anea et al., 2009; Bass and Takahashi, 2010; Evans and Davidson, 2013; Kondratov and Antoch, 2007). In rodents, as well as in humans, there is evidence that sleep disruption leads to neurodegenerative pathology (Benedict et al., 2014; Kang et al., 2009). In humans, common neurodegenerative disorders increase in prevalence with age, and so are becoming more prevalent as the human population ages (Ballard et al., 2011). The primary example, Alzheimer's disease (AD), affects 20-40 million people worldwide, and is the most common cause of progressive cognitive dysfunction (dementia) in adults (Ballard et al., 2011; Prince et al., 2016); it has also been noted to cause circadian dysfunction from an early stage (Osorio et al., 2011; Tranah et al., 2011). Such ageing demographic trends, along with the disruptive effects of the modern environment, such as bright light at night and shift work, could result in a population predisposed to circadian dysfunction (Antunes et al., 2010). This review therefore addresses the question of whether a positive feedback loop exists, in which neurodegenerative disorders are both a cause and an effect of circadian dysfunction.

**Box 1. Glossary**

**Circadian entrainment:** The process by which endogenous oscillations within a period of ∼24 h are synchronized with environmental oscillations. The signal that mediates the entrainment (often light but can also be feeding) is termed the zeitgeber (i.e. time giver or timer).

**Circadian rhythms:** Molecular, hormonal, physiological and behavioral rhythms within a period of ∼24 h.

**Fat body:** This tissue is considered to be the *Drosophila* equivalent of the liver and adipose tissue of vertebrates, in terms of its storage and metabolic functions.

**Glymphatic system:** Clearance pathway for interstitial waste (solute and fluid) in the vertebrate central nervous system.

**Humoral signals:** Signals mediated by hormones.

**Hypothalamus-pituitary-adrenal axis:** Three structures of the endocrine system, namely the hypothalamus, pituitary and adrenal cortex, that constitute the glucocorticoid hormone pathway.

**Neurofibrillary tangles:** Intracellular deposits of the microtubule binding protein tau. Tangle density is correlated with cognitive impairment in AD.

**Rapid eye movement (REM) sleep behaviour disorder:** The loss of normal muscle atonia during REM (dreaming) sleep, resulting in movement, often linked to dream content. This sleep disorder is strongly linked to subsequent development of Parkinson's disease and/or dementia with Lewy bodies.

**Suprachiasmatic nucleus (SCN):** Brain nucleus located in the hypothalamus, above the optic chiasm, which contains the central circadian clock in mammals.

**Tauopathies:** Neurodegenerative diseases associated with the pathological aggregation of the protein, tau, in deposits such as neurofibrillary tangles.

A better understanding of how circadian dysfunction can contribute to neurodegenerative disease mechanisms might help with the development of novel therapies for AD and for other neurodegenerative disorders. There are currently no licensed, disease-modifying treatments for AD, despite enormous efforts aimed primarily at preventing or clearing the characteristic protein deposits that characterize this disease (Table 1). While acknowledging the pathological primacy of amyloid deposition in AD, an understanding of the possible role of circadian disruption in mediating disease progression could provide us with novel therapeutic targets. As circadian mechanisms are highly conserved between flies, rodents and humans, there are a wide range of model systems available for study.

**Table 1.**
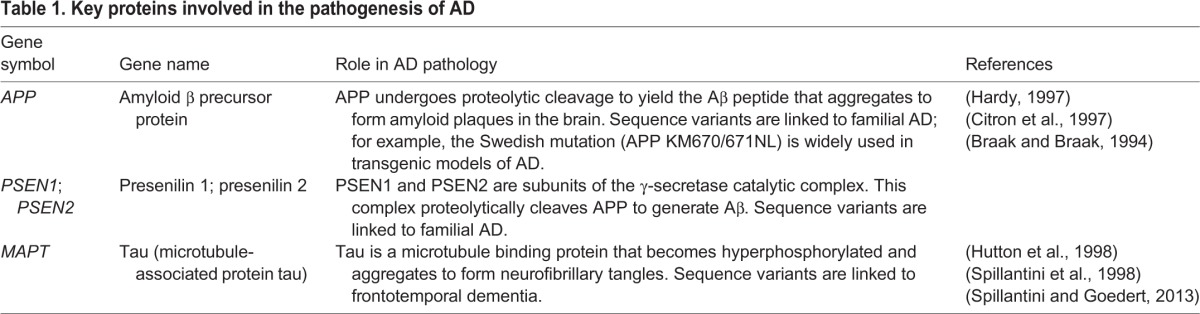
**Key proteins involved in the pathogenesis of AD**

Here, we provide an overview of the molecular and neurological basis of circadian biology in insects and mammals. We discuss evidence from fly and mouse models of AD that highlights the involvement of circadian dysfunction in AD, and shows how circadian dysfunction, specifically sleep disruption, can promote amyloid pathology directly, and disease progression indirectly, through downstream metabolic dysfunction and diabetes. Finally, we discuss a range of therapeutic approaches that aim to correct circadian dysfunction in neurodegenerative diseases, such as AD, including metabolic correction, the restoration of circadian rhythms and the enhancement of sleep.

## The molecular basis of the circadian clock

**Fig. 1.**
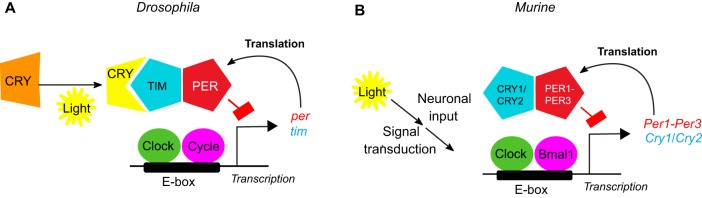
**Conservation of circadian clocks between flies and mice.** The heterodimeric transcription activators, Clock/Cycle in *Drosophila* (A) or Clock/Bmal1 in mice (B) (green and purple ovals, respectively), drive the transcription of the *period*/*timeless* (*per*/*tim*) genes in *Drosophila* (A) and of the genes for the period or cryptochrome proteins (*Per1-Per3* or *Cry1/Cry2*) in mice (B) by binding to the regulatory E-box upstream of target genes. The protein dimers of PER/TIM, PER1-PER3 or CRY1/CRY2 (red and blue pentagons, respectively) in turn negatively regulate Clock/Cycle or Clock/Bmal1 transactivation. This time-delayed, negative feedback is the basis for the temporal oscillation of the molecular clock. Of note, mammalian CRY1/CRY2 and *Drosophila* TIM are functional, not sequence, orthologues. In *Drosophila*, light entrainment can be mediated by CRY, allowing light to directly entrain the molecular clock of both central and peripheral tissues. The cell-autonomous sensing of light by CRY results in the light-dependent degradation of TIM. Such cell-autonomous detection of light is not possible in larger animals as their internal tissues cannot directly sense this zeitgeber.

The molecular basis for circadian rhythms consists of conserved transcriptional and translational feedback loops of so-called ‘clock genes’. In mammals, the core transcriptional machinery consists of the bHLH-PAS [basic helix-loop-helix–PER/aryl hydrocarbon receptor nuclear translocator/single minded homology domain (Kewley et al., 2004)] transcription factors, such as those encoded by the genes *Bmal1* (*Arntl*) and *Clock*. As well as modulating the expression of a vast number of genes across the genome, these factors stimulate the transcription of their own repressors, such as the period (PER1-PER3) and cryptochrome (CRY1/CRY2) proteins. Thus, *Per1-Per3* and *Cry1/Cry2* provide time-delayed inhibition of *Bmal1* and *Clock*, resulting in gene expression patterns that oscillate within a ∼24 h period. Circadian biology has also been studied extensively in the fruit fly, *Drosophila*, because many of the clock genes have orthologues and/or conserved feedback loops (see Fig. 1 for a comparison of the mammalian and *Drosophila* molecular clocks) (Hardin and Panda, 2013; Mohawk et al., 2012).

These clock genes are responsible for circadian oscillations at the cellular level by regulating membrane electrical activity and cellular metabolism (O'Neill and Feeney, 2014; O'Neill and Reddy, 2012). The creation of whole-organism rhythms in behaviour and physiology requires the formation of dedicated neural circuits, made up of cells that express the clock genes within the central nervous system. In mammals, ∼20,000 of such ‘master clock’ neurons reside within the suprachiasmatic nucleus (SCN) (see Glossary, Box 1) of the hypothalamus (Dibner et al., 2010). In *Drosophila*, 150 central clock gene-expressing neurons are subdivided into seven groups that are located in the anterior, posterior and superior brain (Peschel and Helfrich-Förster, 2011).

**Fig. 2.**
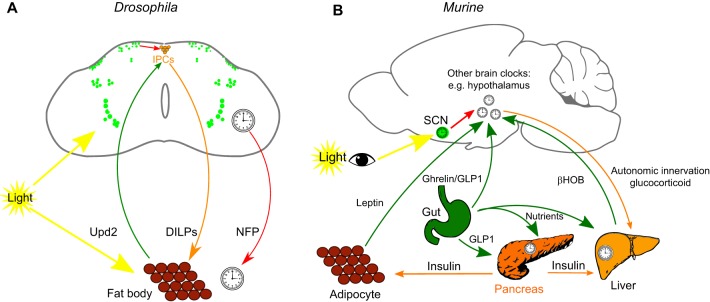
**Entrainment of the central and peripheral clocks in *Drosophila* and mice.** (A) A cross-section of a *Drosophila* brain, dorsal is top. The central clock in *Drosophila* consists of a neuronal network in the brain (green circles). Light directly entrains both the central and peripheral clocks in *Drosophila* via CRY. Peripherally, circadian variation in feeding drives oscillation in stored energy in a *Drosophila* organ called the fat body. The status of energy stores is conveyed to IPCs (orange circles) in the fly brain by the fat body-derived cytokine Upd2 (green arrow). The peripheral clock in the fat body is regulated partly by neuropeptide F (NPF, lower red arrow), which derives from a subset of central clock neurones. Subsets of central clock neurones also regulate the production of *Drosophila* insulin-like peptides (DILPs) in IPCs (top red arrow). DILPs, in turn, regulate circadian oscillations in carbohydrate metabolism in the fat body (orange arrow). (B) A side view of an adult mouse brain, anterior is to the left. In mice, light input is conveyed to the central clock in the SCN (green clock) of the hypothalamus via retinal-hypothalamic neuronal input (yellow arrow). Nutritional status is conveyed to the hypothalamus via gut-derived ghrelin and GLP-1, or by adipocyte-derived leptin (green arrows from gut and adipocytes). Peripheral metabolic clocks are entrained primarily by autonomic innervation and by glucocorticoid hormones (top orange arrow). The food entrained oscillator is likely synchronized by nutrient inputs and by gut-derived hormones (green arrows from gut), such as by GLP1 and insulin (orange arrows from pancreas), peripherally, and by leptin, ghrelin, GLP1 and β-hydroxybutyrate (βHOB), centrally.

While this neural circuitry generates endogenous rhythms within a period of ∼24 h, an environmental cue (a zeitgeber) is also required to keep the organism in synchrony with its optimal temporal niche (Hut et al., 2012). This process is termed circadian entrainment (see Glossary, Box 1). Light is the primary zeitgeber and, as such, is primarily responsible for entraining the endogenous rhythmicity of an organism's neural circuits so that they oscillate in synchrony with their environment (Münch and Bromundt, 2012). In mammals, the predominant mechanism for light entrainment utilizes the nonvisual photoreceptor, melanopsin, which is found in photosensitive retinal ganglion cells that provide input directly to the SCN (Hankins et al., 2008). The central clock communicates with peripheral clocks in other brain regions and in systemic organs, such as the liver, via rhythmic neuronal and humoral signals (see Glossary, Box 1). Unlike its mammalian orthologues, CRY in *Drosophila* acts as a cell-autonomous circadian photoreceptor by destabilising the transcription repressor *timeless* upon light exposure (Fig. 1A) (Koh et al., 2006; Peschel et al., 2009). In this way, light infiltrating the fly's cuticle directly synchronizes the central circadian clock, as well as the peripheral oscillators (Plautz et al., 1997) (see Fig. 2 for a comparison of central and peripheral mammalian and *Drosophila* clock circuitry). Notably, visual photic signals, meaning perceived visual inputs, act as a relatively minor entraining stimulus in mammals (Hankins et al., 2008) and *Drosophila* (Rieger et al., 2003).

Other zeitgebers include nonphotic stimuli, such as temperature, food availability, exercise and social interactions (Buhr et al., 2010; Carneiro and Araujo, 2012; Glaser and Stanewsky, 2007; Hastings et al., 1998; Levine et al., 2002a; Mistlberger and Skene, 2005; Simoni et al., 2014), which under certain circumstances can entrain endogenous rhythmicity. For example, when nocturnal rodents are restricted to a daytime feeding schedule, through the provision of a 2-6 h meal time during their usual rest phase, they exhibit a dissociation of peripheral circadian oscillators from the SCN. Not only will the rodents' activity shift to realign with the expected mealtime, but the timing of clock gene expression in peripheral tissue will also be shifted by the daytime feeding schedule (Boulos and Terman, 1980; Damiola et al., 2000; Stokkan et al., 2001). Meanwhile, the SCN remains entrained to the light-dark cycle under most conditions (Hara et al., 2001).

In *Drosophila*, feeding behaviour is controlled by both central and peripheral clocks, such that feeding rhythms are diminished in flies with either no central clock or with no peripheral clock in the fat body (see Glossary, Box 1) (Xu et al., 2008). In the periphery, the fat body derived cytokine, Unpaired 2 (Upd2), conveys the fed status in *Drosophila* to the insulin-producing cells (IPCs) in the brain (Rajan and Perrimon, 2012). Interestingly, a subset of IPCs has been shown to regulate sleep-wake behaviour in *Drosophila* (Yurgel et al., 2015). Centrally, a subset of clock (DN1) neurons regulates the secretion of insulin-like peptide (ILP) in a circadian pattern, which in turn regulates fat body-mediated sugar homeostasis (Barber et al., 2016) (Fig. 2A).

The concordant and synchronized oscillation of the central clock with the various peripheral tissue clocks is thought to optimally coordinate an organism's metabolism (Bass and Takahashi, 2010), supporting its health and fitness (Nedeltcheva and Scheer, 2014; Roenneberg and Merrow, 2016; Scheer et al., 2009). The desynchronization of the central and peripheral clocks can occur as a result of modern life, as seen in individuals exposed to light-emitting diode (LED) light at night (Hatori et al., 2017) and those undertaking shift work (Kecklund and Axelsson, 2016; Knutsson, 2003; Pan et al., 2011). The aberrant circadian signals in today's environment pose a particular challenge to elderly people who, as we discuss below, exhibit progressively less robust circadian rhythms.

## Circadian clock function in ageing and in age-related disease

Healthy ageing in humans is often linked to changes in the sleep-wake cycle. Typically, older individuals nap more often during the day (Buysse et al., 1992) and experience shallower night-time sleep with more arousals, which disrupt non-rapid eye movement sleep in particular (Dijk et al., 2001). The relative timings (phase relationships) of sleep and of other circadian oscillations, such as body temperature, also change with age (Yoon et al., 2003), likely indicating differences in entrainment communication between various clocks. Data from experimental organisms, such as *Drosophila* (Chen et al., 2014) and mouse (Nakamura et al., 2011), indicate that communication between clock neurons, and between clock neurons and their output pathways, fails earlier than does the circadian cycling of the molecular components of the clock. While *in vitro* (Kunieda et al., 2006) and *in vivo* (Wyse and Coogan, 2010) models of ageing indicate that the molecular clock might also be impaired in aged cells and organisms, the extent to which this contributes to circadian changes in elderly humans (Münch et al., 2005; Schmidt et al., 2012) is unclear. For this reason, model organisms that carry clock gene mutations that abolish molecular rhythmicity, such as mutations in *Bmal1* in mice (Bunger et al., 2000; Laposky et al., 2005) or in *period* (*per^0^*) in flies (Konopka and Benzer, 1971), might not be the optimal models in which to study age-related circadian deficits.

Less robust circadian signalling with age might underpin age-related sleep deficits, which might, in turn, directly injure the brain (Kondratova and Kondratov, 2012). For example, chronic ‘jet lag’ in rodents causes deficits in hippocampal neurogenesis (Rakai et al., 2014) and cognition (Kott et al., 2012), and in long-haul aircrew, jet lag has been linked to reduced hippocampal volume (Cho, 2001). The damage to the hippocampus has been likened to accelerated ageing, likely mediated by astrogliosis and increased production of reactive oxygen species (Ali et al., 2015; Musiek et al., 2013). Such age-related stressors could explain at least some of the increasing incidence of neurodegenerative disease in the elderly.

The neurodegenerative disease we focus on in this review is AD, which is characterized histologically by the dual pathologies of extracellular neuritic (amyloid β, Aβ) plaques (Braak and Braak, 1994) and intraneuronal neurofibrillary tangles (see Table 1 and Glossary, Box 1) (Braak et al., 1994). These pathological features have been replicated to some extent in vertebrate and invertebrate model organisms (Drummond and Wisniewski, 2017; Moloney et al., 2010b). Genetic linkage studies in familial AD (Goate et al., 1991; Rogaev et al., 1995; Sherrington et al., 1995) and whole-genome studies of the more common, sporadic form of AD (Jonsson et al., 2012) indicate that the increased production of aggregation-prone Aβ peptides, the main component of plaques (Glenner and Wong, 1984; Masters et al., 1985a,b), might initiate the disease. In addition, genome-wide association studies have implicated a wider range of biological functions that likely contribute to risk of AD, in particular innate immunity and inflammation (Cuyvers and Sleegers, 2016; Guerreiro et al., 2013; Jonsson et al., 2013; Lambert et al., 2013; Singleton and Hardy, 2016).

Memory deficits are a cardinal symptom of AD. However, many individuals with AD experience an earlier symptom (prodrome) characterized by disrupted sleep; this likely explains the strong link between ever taking benzodiazepine sleeping medication and risk of dementia (Billioti de Gage et al., 2014). In established AD, the main sleep abnormalities resemble an exaggerated form of the sleep changes that occur during healthy ageing. The main features include increased night-time wakefulness, caused by increased sleep latency and reduced sleep consolidation (the duration of uninterrupted sleep episodes), reduced slow-wave sleep and increased day-time naps (Bonanni et al., 2005; Musiek et al., 2015). Additionally, individuals with moderate and advanced AD may exhibit ‘sundowning’, where agitation is more marked in the late afternoon (Bedrosian and Nelson, 2013; Volicer et al., 2001). Circadian disturbance is also evident in the daily rhythms of activity and core body temperature. Individuals with AD typically show two abnormalities: first, there is less differentiation between day and night and second, the oscillations are phase shifted so that peaks in both body temperature and activity occur later in the day as compared to healthy controls (Harper et al., 2001; Satlin et al., 1995; Tranah et al., 2011; Volicer et al., 2001). By contrast, men with frontotemporal dementia exhibit activity oscillations that are phase advanced as compared to AD (Harper et al., 2001).

The link between sleep disorders and Parkinson's disease is arguably even stronger, with rapid eye movement (REM) sleep behaviour disorder (see Glossary, Box 1) preceding classical Parkinsonian features by decades in some instances (Postuma and Berg, 2016). As many as 90% of individuals diagnosed with REM sleep behaviour disorder will go on to develop a neurodegenerative disease associated with α-synuclein aggregation (Boeve et al., 2001; Iranzo et al., 2014). The mechanisms underlying this exceptionally high rate of association are unknown; however, this association supports the hypothesis that the neural circuits controlling sleep-wake behaviour are particularly vulnerable in the early stages of neurodegenerative disease.

## Circadian disorders in AD models: role of the central clock

**Fig. 3.**
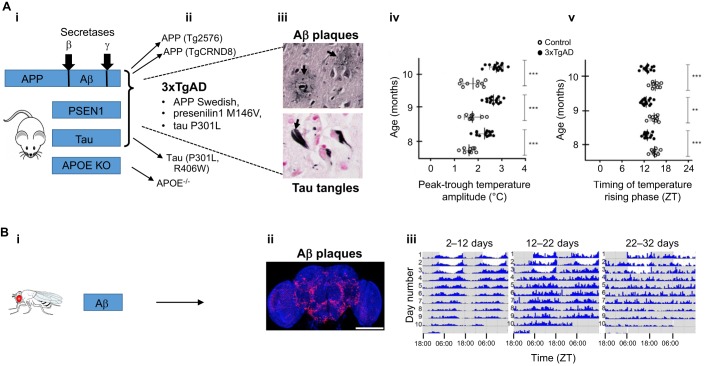
**Mouse and *Drosophila* models of AD.** (A) (i) Murine models of AD are typically generated by the overexpression of disease-linked variants of human proteins, such as APP, the active subunit of γ-secretase (PSEN1) and/or tau. The APOE knockout (KO) mouse also serves as a model of AD. (ii) Tg2576 (Hsiao et al., 1996) and TgCRND8 (Janus et al., 2000) mice express mutants of human APP that are cleaved by β- and γ-secretases to generate neurotoxic Aβ peptides. The APP transgene can be combined with other transgenes, as for example in the 3×TgAD mouse, which carries human disease-linked variants of APP (the so-called Swedish mutation), PSEN1 and tau (Oddo et al., 2003). This triple transgenic mouse replicates many features of AD (see Aβ plaques and tau tangles in iii, arrows). High power views of Gallyas silver-stained Aβ plaques (image credit: Jensflorian, Wikimedia Commons) and Gallyas silver-stained tau tangles (image credit: Patho, Wikimedia Commons). Tauopathy, a neurodegenerative disorder related to AD, can be modelled in P301L (Lewis et al., 2000) and R406W (Tatebayashi et al., 2002) tau transgenic mice. APOE KO mice might replicate some features of AD, such as amyloid and tau deposition, and exhibit metabolic and circadian abnormalities (Zhou et al., 2016), although these observations require independent replication. The 3×TgAD mice also exhibit mild circadian dysfunction, including differences in the amplitude (iv) and timing (v) of body temperature oscillations (Knight et al., 2013). (B) (i) Fruit fly models of Aβ toxicity are typically generated by expressing the Aβ peptide downstream of a secretion signal peptide (Crowther et al., 2005; Finelli et al., 2004; Iijima et al., 2004). (ii) In these models, intraneuronal and extracellular deposits of Aβ are visible in transverse sections of fly brain stained with anti-Aβ antibodies (red, cell nuclei in blue; reproduced with permission from Ott et al., 2015). Scale bar: 200 µm. (iii) The expression of toxic forms of the Aβ_1-42_ peptide, such as the E22G Arctic variant, elicit progressive sleep deficits as evidenced by the loss of the rhythmicity in the actimetry traces as the transgenic flies age (reproduced with permission from Chen et al., 2014). Wild-type flies retain a youthful pattern of behaviour, resembling the 2-12 days data, at all time points shown.

Murine transgenic models of AD have been generated in various ways to yield human-like AD pathology (Fig. 3). In one approach, extracellular amyloid pathology has been driven by expressing disease-linked variants of the human Aβ precursor protein (*APP*) (Table 1). Such transgenes can be combined with disease-linked variants of human presenilin 1 (*PSEN1*) (Table 1), which encodes a subunit of the complex that cleaves APP to generate the Aβ peptide. Mice have also been generated to express variants of human tau, for example P301L or R406W, that are linked to human tauopathies (see Table 1 and Glossary, Box 1), such as frontotemporal dementia. The circadian consequences of overexpressing APP or Aβ include abnormalities in sleep, locomotor and body temperature rhythms (Ambrée et al., 2006; Gorman and Yellon, 2010; Wisor et al., 2005). Interestingly, mice that express disease-associated variants of human *APP*/*PSEN1* also exhibit phase delays similar to those identified in patients with AD (Duncan et al., 2012). By contrast, mice that express either of two human tau variants that contain disease-linked substitutions (P301L and R406W) exhibit abnormalities in a sleep electroencephalogram but no disruption to their circadian rhythms (Koss et al., 2016). The triple-transgenic mouse model (which carries three human variants associated with AD: the *APP* Swedish variant, KM670/671NL, the *PSEN1* variant, M146V, and the *MAPT* variant, P301L) exhibit decreased nocturnal activity (the equivalent of daytime napping in a nocturnal species), increased daytime activity (Sterniczuk et al., 2010) and age-related changes in body temperature rhythms (Knight et al., 2013). These mice also exhibit reductions in the number of vasoactive intestinal polypeptide- and arginine vasopressin-containing neurons that constitute the central clock mechanism (Sterniczuk et al., 2010). SCN degeneration and dysfunction have been observed in an apolipoprotein E (APOE) knockout mouse that recapitulates several aspects of human AD (Zhou et al., 2016), although these findings have not been replicated by other groups as yet and should thus be interpreted with caution. These findings indicate that specific neurodegenerative lesions in the SCN might cause circadian deficits, a hypothesis that finds some support in human histopathological studies (Swaab et al., 1988; Zhou et al., 1995). However, amyloid plaques are relatively sparse in the SCN in AD (Stopa et al., 1999), excluding bulk Aβ deposition as the cause of neurodegeneration. Perhaps smaller, soluble Aβ species are responsible instead, as suggested by the transplantation of PC12 cells that express a disease-linked *APP* variant into rats; the transplantation of these cells (but not of control PC12 cells) causes circadian deficits (Tate et al., 1992). Despite the relatively mild circadian deficits observed in murine models of AD (Coogan et al., 2013), one study has shown that in a knock-in mouse model of human *APP*/*PSEN1* genes, the mice have a reduced amplitude of endogenous *Per2* mRNA oscillation in the SCN (Duncan et al., 2012). In *Drosophila*, there have been similar findings, in particular the boosting of the cleavage of APPL (the *Drosophila* orthologue of APP) by β-secretase resulted in behavioural arrhythmia and reduced the expression of the clock gene *per* (Blake et al., 2015). However, fly models of human Aβ toxicity do not point to oscillatory failure in the central clock as the primary cause of circadian dysfunction. Instead, the data, which are also supported by similar findings in mammals, point to defects in the clock output pathways, as discussed in more detail in the next section.

## Central clock output failure in neurodegenerative disease

Vertebrate and invertebrate models of neurodegenerative disease have shown that robustly rhythmic central molecular clocks can become disconnected from other brain-resident and peripheral clocks, to result in disrupted circadian behaviour. In particular, mouse and *Drosophila* models of Huntington's disease (Pallier et al., 2007) and AD (Chen et al., 2014; Khabirova et al., 2016; Long et al., 2014) exhibit normal central clock function. Despite this, they exhibit behavioural arrhythmia, including disrupted sleep consolidation and the sleep/wake cycle (Khabirova et al., 2016). In the case of the R6/2 mouse model of Huntington's disease, the mice were behaviourally arrhythmic and had severely disrupted sleep-wake cycles, and yet the electrophysiological activity of acute SCN brain slices from mutant mice was normal (Pallier et al., 2007). Moreover, the molecular clock in the SCN remained essentially intact in these mice, as recorded using an mPer1::luciferase bioluminescence reporter construct. Although upstream factors could affect the function of the SCN, the authors concluded that the results were consistent with a failure of the SCN to entrain downstream oscillators (Maywood et al., 2010; Pallier et al., 2007). Similarly, *Drosophila* that express human Aβ as a model of AD show progressive behavioural arrhythmia, despite the essentially normal oscillation of their central molecular clock (as shown by the use of a luciferase reporter) (Chen et al., 2014). These behavioural deficits were accompanied by the disruption of peptidergic neurones and of the synapses that mediate the output from the central clock.

Such findings in experimental animals are complemented by human post-mortem studies, which have compared the brains of individuals with and without a diagnosis of AD. For example, in human brain tissue from individuals with AD post mortem, the expression of clock genes in the pineal, a structure that receives central clock inputs, was found to be similar to the gene expression changes seen in rats in which the SCN-pineal projection had been experimentally lesioned (Wu et al., 2006). This suggests that in the AD brains examined, the pineal gland was deprived of its normal entraining input, which is notable because of the role of this gland in the secretion of the sleep associated hormone, melatonin. Additionally, Cermakian and colleagues measured clock gene expression in various human brain structures and correlated expression levels with the time of each individual's death. Their conclusion was that the central, and indeed the secondary, brain clocks were rhythmic in healthy individuals and in those with AD, but in the latter there were marked phase shifts, indicating changes in their relative synchronization (Cermakian et al., 2011). This failure of clock synchronization is caused by deficits, likely at the synaptic level, in the communication of entrainment signals between clocks. One consequence of disrupting the various circadian oscillators in the brain is that sleep, the most easily accessible circadian phenotype, is affected early and profoundly. While sleep disruption in AD has been documented for decades, how this condition links mechanistically to the molecular pathogenesis of AD has become apparent only relatively recently, as discussed below.

## Sleep disruption and risk of amyloid pathology

As recounted above and elsewhere (Holth et al., 2017; Ju et al., 2014; Musiek and Holtzman, 2016; Musiek et al., 2015), neurodegenerative disease results in the loss of restorative sleep, which might in turn accentuate the pathological processes that contribute to AD. This view is partly based on studies that show that diurnal fluctuations in Aβ levels in the cerebrospinal fluid (CSF) and interstitial fluid (ISF) are directly associated with sleep-wake behaviour in both mice and humans. For example, Aβ in the ISF is higher during wakefulness in mice, representing periods of peak neuronal activity (Bero et al., 2011), and lowest during sleep (Huang et al., 2012; Kang et al., 2009) or under anaesthesia (Brody et al., 2008). In humans, this circadian variability in Aβ levels declines with age and with the progression of AD pathology (Huang et al., 2012). Sleep restriction exacerbates protein deposits in both the APP/PSEN1 (Kang et al., 2009) and the triple transgenic (3×TgAD) mouse models (Di Meco et al., 2014; Rothman et al., 2013). In healthy humans, even acute sleep deprivation is sufficient to cause detectable neuronal damage, as reflected by the presence of markers of neuronal and of blood-brain barrier damage in the blood of healthy volunteers (Benedict et al., 2014).

One factor that might contribute to the circadian variation of Aβ levels is the 60% expansion in ISF volume that occurs in the mouse brain during sleep (Xie et al., 2013). Similar changes in the human brain during sleep would favour the bulk flow of CSF and ISF through the perivascular (Rennels et al., 1985) and glymphatic drainage channels (Iliff et al., 2012; Kress et al., 2015; Tarasoff-Conway et al., 2015). The glymphatic system (see Glossary, Box 1) would then deliver CSF and ISF solutes, including Aβ, to the cervical lymph nodes for disposal in the periphery. The structural integrity of the glymphatic channels, as reported by the polarized perivascular expression of aquaporin 4 (*AQP4*), declines with age and more so when accompanied by amyloid pathology (Zeppenfeld et al., 2017). AQP4 likely mediates water influx into the glymphatic system, facilitating its flow, and is itself under circadian control (Zuber et al., 2009).

Circadian oscillations in the levels of Aβ are mirrored by orexin, a hormone released from neurones in the hypothalamus (de Lecea et al., 1998). Orexin promotes wakefulness, and loss of its signalling causes narcolepsy, a disorder of unwanted sleep intrusions (Pintwala and Peever, 2017). Orexin knockout mice sleep more than controls (Chemelli et al., 1999), and when crossed with APP/PSEN1 mice, they exhibit a marked reduction in Aβ plaque deposition (Roh et al., 2014). In the resulting APP/PSEN1/Or (Hcrt)^−/−^ mice, lentiviral-mediated expression of orexin in the hippocampus failed to rescue the amyloid pathology, indicating that orexin does not have a direct action on susceptible neurones. By contrast, orexin expression in the hypothalamus, or indeed sleep deprivation, made amyloid pathology worse in the APP/PSEN1/Or^−/−^ mice, as compared to mice injected with control lentivirus, or mice that were not sleep-deprived (Roh et al., 2014). This benefit of orexin blockade was confirmed in the Tg2576 AD mouse model, in which treatment with an orexin receptor blocker, almorexant, suppressed the normally elevated nocturnal levels of Aβ and reduced plaque accumulation (Kang et al., 2009). Although these effects are striking, the data do not conclusively show that sleep itself is protective. In this regard, sleep-inducing GABA agonists have been shown to improve cognitive dysfunction in *Drosophila* that express presenilin variants linked to AD in humans (Dissel et al., 2015). A GABA agonist had similar benefits in the R6/2 mouse model of Huntington's disease (Pallier et al., 2007).

Taken together, the combination of reduced Aβ production, increased Aβ clearance and an increase in the volume of ISF makes sleep a valuable process for the prevention, and clearance, of protein aggregates, thereby reducing the risk of neurodegenerative disease. However, the impact of circadian dysfunction reaches beyond the central components of the circadian clock. As we discuss below, the breakdown of peripheral metabolic rhythms might also contribute materially to the pathogenesis of AD.

## Peripheral clock arrhythmia and diabetes

**Fig. 4.**
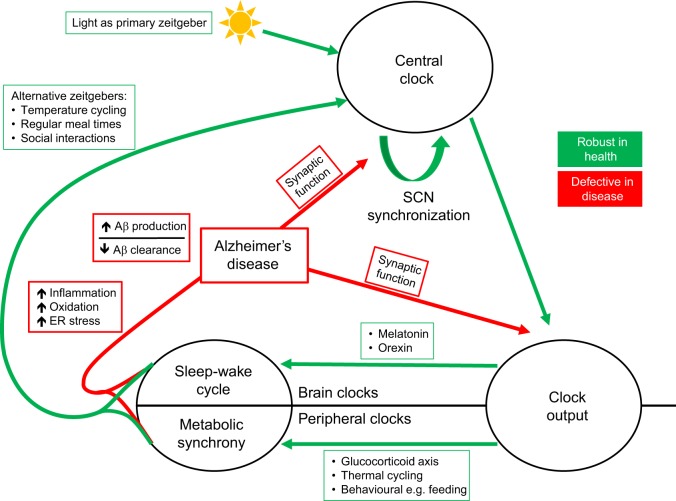
**A model of how circadian biology and AD pathology might interact.** Interactions between the central clock and other brain and peripheral clocks occur via the clock output pathway. Subsequent metabolic, behavioural and social cycles ensure the optimal functioning of the organism and might feedback to entrain the central clock, alongside nonvisual light input (which is the primary zeitgeber). Disrupted sleep and metabolic asynchrony might predispose an individual to AD pathology. This, in turn, might accentuate circadian deficits by damaging synaptic and other functions in the central clock mechanism and output pathway. ER stress, endoplasmic reticulum stress caused by protein misfolding and aggregation.

Disturbed clocks in the brain have deleterious consequences for the whole organism, disrupting directly, or indirectly, the concerted hormonal, autonomic and metabolic functioning of various organ systems (Delezie and Challet, 2011; Reddy and Maywood, 2007) (Fig. 4). The negative effects of chronic circadian misalignment is evident in shift workers, who have an increased risk for obesity, type 2 diabetes and cardiovascular disease (Antunes et al., 2010; Haus and Smolensky, 2006; Pan et al., 2011; Scheer et al., 2009).

Central to metabolic health is the synchronization of gut, liver and muscle metabolic cycles and their interplay with the glucocorticoid hormones secreted by the hypothalamus-pituitary-adrenal axis (see Glossary, Box 1) (Dickmeis, 2009; Gamble et al., 2014; Reddy et al., 2007). In health, a host of hepatic genes, including many involved in metabolism (Schmutz et al., 2010), exhibit circadian transcriptional regulation (Delezie and Challet, 2011). However, most require clock mechanisms local to the liver to sustain their oscillations, rather than relying entirely on signals from the SCN. This was demonstrated in a mouse model in which the liver clock was specifically suppressed, resulting in all but a handful of genes losing their circadian regulation (Kornmann et al., 2007). By contrast, in the presence of functioning peripheral clocks, centrally derived signals, such as glucocorticoid hormones, are sufficient to entrain efficiently most circadian gene expression in tissues (Reddy et al., 2007). In entrained mice, the subsequent loss of central clock signals, for example by experimental lesioning of the SCN, does not destroy tissue-specific peripheral clocks; rather they continue to function but become progressively desynchronized, both between tissues within one animal, and between animals (Yoo et al., 2004).

The desynchronization of peripheral clocks may also be induced by feeding rodents during the day, when they are normally sleeping. For example, Yasumoto and colleagues found that the daytime feeding of mice with a high fat and high sucrose diet resulted in the desynchronization of peripheral clocks, as measured by a range of hormones and metabolites that normally show circadian oscillation. The loss of synchrony occurs as different tissues re-entrain to the new feeding schedule at different rates. At the end of the week-long study, the daytime-fed mice gained more adipose tissue, were less physically active, exhibited increased levels of plasma insulin, and accumulated more triglycerides and cholesterol in their liver as compared to mice fed the same diet but during their active phase (Yasumoto et al., 2016). Such outcomes resemble the features of the metabolic syndrome (Sperling et al., 2015), characterized in humans by insulin resistance, abdominal obesity, abnormal lipids and hypertension, which is linked to type II diabetes. In humans, poor sleep patterns, even in the absence of overt neurodegenerative disease, are a risk factor for the metabolic syndrome and for subsequent type II diabetes (Bass and Takahashi, 2010; Pan et al., 2011; Spiegel et al., 2009; Yaggi et al., 2006).

In population-based studies, diabetes is an established risk factor for accelerated age-related cognitive decline (Allen et al., 2004), for dementia as a syndrome (Biessels et al., 2006) and for AD in particular (Huang et al., 2014; Kopf and Frölich, 2009; Wang et al., 2012). Indeed, individuals with type II diabetes who also carry the ε4 *APOE* allele are over five times more likely to develop AD than are those with neither diabetes nor the ε4 allele (Peila et al., 2002). Post-mortem human studies have indicated that the insulin resistance that occurs peripherally in type II diabetes is also seen in the brain of AD patients (Kleinridders, 2016; Talbot et al., 2012). In particular, several studies have found that insulin receptor and also insulin-like growth factor 1 receptor are expressed at lower levels on the surface of neurons in the brains of individuals with AD. These changes are accompanied by phosphorylation of the corresponding signalling proteins, such as the insulin receptor substrate 2 (Irs2), which is a hallmark of suppressed insulin signalling (Holscher, 2014a; Kleinridders, 2016; Moloney et al., 2010a; Rivera et al., 2005; Steen et al., 2005; Talbot et al., 2012). The downregulation of Irs2 signalling has been modelled by knocking out the *Irs2* gene in the mouse. The resulting animals show decreased brain size accompanied by an increase in tau phosphorylation (Schubert et al., 2003), an observation that is concordant with the tau hyperphosphorylation observed in the brains of AD patients. In the mouse, elevated glucose levels are observed to interact with both age and Aβ pathology by increasing the levels of the Aβ peptide in the hippocampal ISF; the induced Aβ elevation was most marked in aged mice that had extensive plaque pathology (Macauley et al., 2015).

In diabetes, Aβ pathogenesis might also be enhanced as a consequence of endoplasmic reticulum (ER) stress (Cretenet et al., 2010; Maillo et al., 2017), and by the associated oxidative and glycation damage that promotes a neuroinflammatory response, which is likely mediated by activated microglia (Catrysse and van Loo, 2017; Villegas-Llerena et al., 2016). Both the presence of amyloid aggregates and the dyslipidaemia associated with diabetes might trigger the toll-like receptor 4 (TLR4) receptor. Activating this mediator of innate immunity, which classically responds to bacteria-derived lipopolysaccharide, likely enhances the proinflammatory environment in the brain in AD (Balducci et al., 2010; Huang et al., 2017). These pathological concepts have been tested *in vivo* by crossing obese (*ob/ob*, also known as *Lep^ob^/Lep^ob^*) mice, which develop a diabetic phenotype due to the leptin gene mutation, with *App* mutant mice. The APP^+^-*ob/ob* mice have more severe cognitive deterioration, neuroinflammation and more rapid amyloid deposition in the cerebral vasculature than either of the parental mouse strains (Takeda et al., 2010).

The concept of AD as a form of diabetes in the brain is further supported by observations of cerebral glucose hypometabolism from the earliest stages of AD. For example, individuals carrying genetic risk factors for AD exhibit lower levels of ^18^F-fluorodeoxyglucose uptake in the cortex during positron emission tomography several years before the onset of clinical symptoms (Cunnane et al., 2011). In this context, recent advances in metabolic medicine using, for example, glucagon-like peptide 1 (GLP1, also known as GCG) analogues to reverse insulin resistance and to reduce neuroinflammation, should be further investigated in the context of neurodegenerative disease (Aviles-Olmos et al., 2014; Holscher, 2014b).

If such metabolic dysfunction does indeed occur as a consequence of circadian desynchrony, a useful therapeutic approach might be to resynchronize the central and peripheral clocks. As discussed in the following section, an attractive strategy to resynchronize circadian oscillations is to provide novel zeitgebers or to enhance existing ones.

## Re-entraining circadian clocks: light therapy and other zeitgebers

Circadian clocks might be amenable to direct intervention in order to benefit individuals living with AD. Considering the primary importance of photic entrainment of the SCN, initial studies employed light as a therapy, typically providing a bright environment during the day and usually combined with darkness at night (Fetveit et al., 2003; Lack et al., 2005; Pallesen et al., 2005; Sharkey et al., 2011). As a recent meta-analysis confirmed, light therapy is effective in improving sleep-wake deficits, at least in women; however, the effect sizes are small (van Maanen et al., 2016). If the output of the SCN were defective in AD, then this result would be expected since the light-entrained central clock would be functional but (as discussed above) unable to communicate effectively with other brain clocks and with the periphery.

The disappointing efficacy of light therapy has raised interest in other, nonphotic, zeitgebers that target peripheral circadian clocks; potential candidates include temperature (Buhr et al., 2010; Glaser and Stanewsky, 2007), food availability (Carneiro and Araujo, 2012), exercise (Atkinson et al., 2007; Edgar and Dement, 1991; Miyazaki et al., 2001) and social interactions (Hastings et al., 1998; Levine et al., 2002b; Mistlberger and Skene, 2005; Simoni et al., 2014). Although each of these zeitgebers offers a potential intervention, entrainment to a regular feeding regimen is particularly promising. This is because a so-called food-entrained oscillator (FEO) can act as an alternative master clock, driving circadian sleep and behavioural activity. Evidence of the FEO power as an entraining factor has come from experiments in rodents in which daytime feeding, which is antiphase in a nocturnal species, was sufficient to entrain the animals to a new, anticipatory sleep/wake cycle (Carneiro and Araujo, 2012). Once entrained, the rats continue to wake early even when no food is provided and adapt only gradually to new patterns of food provision, features that are characteristic of an entrained circadian clock. Remarkably, this entrainment is still possible in rats with SCN lesions, leading to the proposal that the FEO has distinct neurological components. However, beyond establishing that this oscillator is located outside the SCN, the neurological basis for the clock and its entrainment signals have yet to be determined.

There is also evidence that the FEO can re-entrain model organisms that have been rendered arrhythmic by neurodegenerative processes. In particular, Maywood and colleagues have shown progressive deficits in sleep/wake rhythms in the R6/2 mouse model of Huntington's disease (Maywood et al., 2010). In these mice, there is a concomitant loss of the rhythmic expression of genes involved in liver clock function and metabolism; however, intrinsic clock function remains intact in *ex vivo* cultures of liver and of other tissue slices from R6/2 mice. Thus, in the aged R6/2 mouse, the liver is competent to exhibit circadian oscillations in gene expression but fails to do so. This is probably because of the loss of the central entrainment signal from the SCN and also because of the chaotic dietary signals generated by arrhythmic feeding patterns. Indeed, in the same study, a temporally restricted feeding regimen successfully restored circadian behavioural and hepatic rhythms in aged R6/2 mice (Maywood et al., 2010). Indeed, in studies of wild-type aged mice exhibiting mild metabolic desynchrony, Tahara and colleagues have shown that the FEO might provide a more potent entraining signal for peripheral tissues than the SCN (Tahara et al., 2017). Remarkably, similar studies in ageing *Drosophila* have shown that daytime-only feeding consolidates the sleep/wake cycle and slows age-related degeneration, at least in cardiac function (Gill et al., 2015).

Our understanding of the mechanisms that regulate and operate the FEO is incomplete. However, metabolic hormones such as glucocorticoids, ghrelin, leptin, insulin, glucagon and glucagon-like peptide-1 (GLP1), which exhibit daily rhythms of synthesis and secretion, are all proposed zeitgebers for circadian oscillators (Gil-Lozano et al., 2014; Patton and Mistlberger, 2013). There is some evidence that leptin and ghrelin can modulate food-entrained rhythms, acting peripherally but also through feedback to hypothalamic circuits in the brain (Escobar et al., 2009; Lockie, 2013). In this context, pharmacological gut peptide agonists could act as novel zeitgebers and might offer opportunities to entrain circadian rhythms when light and melatonin therapies fail.

Recently, liver-derived beta-hydroxybutyrate (βOHB) was identified as an important nonphotic zeitgeber in a study that used mice with liver- and brain-specific *Per2* deletions (Chavan et al., 2016). βOHB is the most abundant circulating ketone body and serves as an alternative energy source for tissues, including the brain, when glucose levels are low (Newman and Verdin, 2014). Exploring the utility of βOHB as a potential nonphotic zeitgeber could be particularly valuable for AD, given the deficit in brain glucose metabolism noted above (Cunnane et al., 2011).

## Sleep enhancement as therapy for AD

Sedative drugs have long been the mainstay for improving sleep/wake rhythms in patients with neurodegenerative disorders. The most common treatments include GABA agonists and sedating antidepressants, such as Trazodone, and antihistamines; in difficult cases, atypical antipsychotics can be used (McCleery et al., 2016). However, these treatments have unwanted effects, including excessive daytime sleepiness, anticholinergic effects, such as mouth dryness and urinary retention, and, in the case of antipsychotics, increased mortality (Camargos et al., 2011, 2012, 2014; Kales et al., 2012; McCleery et al., 2016). Trazodone might also have an unexpected beneficial role in suppressing excessive ER stress signaling, at least in murine models of prion disease and tauopathy (Halliday et al., 2017). Alternative approaches have sought to intervene at the level of the circadian signals that are thought to control, or at least to consolidate, sleep rhythms. In this regard, melatonin has been trialled as a therapeutic for AD because of its use, with uncertain clinical evidence, for treating insomnia and jetlag in otherwise healthy individuals (Costello et al., 2014; Pandi-Perumal et al., 2007). Melatonin is secreted from the pineal gland, beginning in the early evening and reaching peak concentrations soon after midnight (Wehr et al., 2001). In the zebrafish, it is essential for synchronising the central clock with sleep rhythms, as demonstrated by Gandhi and colleagues. These researchers knocked out the zebrafish gene that encodes the melatonin biosynthesizing enzyme, aanat2, which resulted in the complete loss of sleep/wake rhythms when the fish were placed in constant darkness (Gandhi et al., 2015). Unfortunately, these insights have not translated well into the clinic because therapeutic trials of melatonin have yielded little to no improvement in the sleep of individuals with AD (McCleery et al., 2016; Xu et al., 2015). These data indicate that abnormal melatonin secretion in AD is not the primary cause of AD-associated sleep abnormalities.

Orexin, as discussed earlier, is a hormone that promotes wakefulness. In rodent models of AD, almorexant, an antagonist that blocks both the OXR1 and OXR2 (HCRTR1 and HCRTR2) orexin receptors, reduces amyloid pathology (Kang et al., 2009). Concordant with these observations, Liguori and colleagues found that individuals with AD had elevated levels of orexin over controls, and that these increased levels correlated positively with both sleep deficits and cognitive decline (Liguori et al., 2014). In our view, these data may provide ample biological justification for future trials of orexin antagonists as a therapeutic for AD.

## Conclusion

Circadian biology and the sleep cycle are disrupted in a number of neurodegenerative diseases but the precise reasons for this remain unknown. Nevertheless, pathology within the central clock, and the impairment of its communication with peripheral clocks, are likely to be important factors contributing to circadian dysfunction in these diseases. The changes in sleep and feeding rhythms that occur as a result of neurodegenerative disease predispose the brain to the pathological processes that contribute to AD and to other neurodegenerative disorders. Important predisposing factors include reduced protein clearance from the brain, and central-peripheral metabolic desynchrony, which likely contributes to the prevalence of the metabolic syndrome and/or diabetes. Thus, circadian disruption in AD can be seen as both a cause and an effect of neurodegeneration.

Interventions that aim to re-entrain the central clock using light have largely failed and so other therapeutic avenues are now being investigated. The FEO is a promising target that might be susceptible to environmental and/or to pharmacological interventions. All clinical trials in AD are likely to be lengthy and costly; however, a trial of simple dietary interventions that maintain a clear circadian rhythm in individuals with early AD is feasible and should be pursued. Pharmacological simulation of entraining signals, either photic or dietary, have not been developed but might have utility. The direct enhancement of sleep by modulating physiological regulators, such as orexin, might also offer advantages over previous hypnotic and antipsychotic approaches. Orexin antagonists are already being investigated for treatment of primary insomnia (Kishi et al., 2015); perhaps now they can be trialled for the bigger prize of disease modification in neurodegenerative disease.

## References

[DMM030627C1] Ali A. A. H., Schwarz-Herzke B., Stahr A., Prozorovski T., Aktas O. and von Gall C. (2015). Premature aging of the hippocampal neurogenic niche in adult Bmal1-deficient mice. *Aging (Albany. NY).* 7, 435-449. 10.18632/aging.100764 26142744 PMC4505169

[DMM030627C2] Allen K. V., Frier B. M. and Strachan M. W. J. (2004). The relationship between type 2 diabetes and cognitive dysfunction: longitudinal studies and their methodological limitations. *Eur. J. Pharmacol.* 490, 169-175. 10.1016/j.ejphar.2004.02.054 15094083

[DMM030627C3] Ambrée O., Touma C., Görtz N., Keyvani K., Paulus W., Palme R. and Sachser N. (2006). Activity changes and marked stereotypic behavior precede Abeta pathology in TgCRND8 Alzheimer mice. *Neurobiol. Aging* 27, 955-964. 10.1016/j.neurobiolaging.2005.05.009 15993515

[DMM030627C4] Anea C. B., Zhang M., Stepp D. W., Simkins G. B., Reed G., Fulton D. J., Rudic R. D. and Daniel Rudic R. (2009). Vascular disease in mice with a dysfunctional circadian clock. *Circulation* 119, 1510-1517. 10.1161/CIRCULATIONAHA.108.827477 19273720 PMC2761686

[DMM030627C5] Antunes L. C., Levandovski R., Dantas G., Caumo W. and Hidalgo M. P. (2010). Obesity and shift work: chronobiological aspects. *Nutr. Res. Rev.* 23, 155-168. 10.1017/S0954422410000016 20122305

[DMM030627C6] Atkinson G., Edwards B., Reilly T. and Waterhouse J. (2007). Exercise as a synchroniser of human circadian rhythms: an update and discussion of the methodological problems. *Eur. J. Appl. Physiol.* 99, 331-341. 10.1007/s00421-006-0361-z 17165050

[DMM030627C7] Aviles-Olmos I., Dickson J., Kefalopoulou Z., Djamshidian A., Kahan J., Ell P., Whitton P., Wyse R., Isaacs T., Lees A. et al. (2014). Motor and cognitive advantages persist 12 months after exenatide exposure in Parkinson's disease. *J. Parkinsons. Dis.* 4, 337-344. 10.3233/JPD-140364 24662192

[DMM030627C8] Balducci C., Beeg M., Stravalaci M., Bastone A., Sclip A., Biasini E., Tapella L., Colombo L., Manzoni C., Borsello T. et al. (2010). Synthetic amyloid-beta oligomers impair long-term memory independently of cellular prion protein. *Proc. Natl. Acad. Sci. USA* 107, 2295-2300. 10.1073/pnas.0911829107 20133875 PMC2836680

[DMM030627C9] Ballard C., Gauthier S., Corbett A., Brayne C., Aarsland D. and Jones E. (2011). Alzheimer's disease. *Lancet* 377, 1019-1031. 10.1016/S0140-6736(10)61349-9 21371747

[DMM030627C10] Barber A. F., Erion R., Holmes T. C. and Sehgal A. (2016). Circadian and feeding cues integrate to drive rhythms of physiology in Drosophila insulin-producing cells. *Genes Dev.* 30, 2596-2606. 10.1101/gad.288258.116 27979876 PMC5204352

[DMM030627C11] Bass J. and Takahashi J. S. (2010). Circadian integration of metabolism and energetics. *Science* 330, 1349-1354. 10.1126/science.1195027 21127246 PMC3756146

[DMM030627C12] Bedrosian T. A. and Nelson R. J. (2013). Sundowning syndrome in aging and dementia: research in mouse models. *Exp. Neurol.* 243, 67-73. 10.1016/j.expneurol.2012.05.005 22627081

[DMM030627C13] Benedict C., Cedernaes J., Giedraitis V., Nilsson E. K., Hogenkamp P. S., Vågesjö E., Massena S., Pettersson U., Christoffersson G., Phillipson M. et al. (2014). Acute sleep deprivation increases serum levels of neuron-specific enolase (NSE) and S100 calcium binding protein B (S-100B) in healthy young men. *Sleep* 37, 195-198. 10.5665/sleep.3336 24470708 PMC3902870

[DMM030627C14] Bero A. W., Yan P., Roh J. H., Cirrito J. R., Stewart F. R., Raichle M. E., Lee J.-M. and Holtzman D. M. (2011). Neuronal activity regulates the regional vulnerability to amyloid-β deposition. *Nat. Neurosci.* 14, 750-756. 10.1038/nn.2801 21532579 PMC3102784

[DMM030627C15] Biessels G. J., Staekenborg S., Brunner E., Brayne C. and Scheltens P. (2006). Risk of dementia in diabetes mellitus: a systematic review. *Lancet Neurol.* 5, 64-74. 10.1016/S1474-4422(05)70284-2 16361024

[DMM030627C16] Billioti de Gage S., Moride Y., Ducruet T., Kurth T., Verdoux H., Tournier M., Pariente A., Begaud B. and Bégaud B. (2014). Benzodiazepine use and risk of Alzheimer's disease: case-control study. *BMJ* 349, g5205-g5205 10.1136/bmj.g5205 PMC4159609 25208536

[DMM030627C17] Blake M. R., Holbrook S. D., Kotwica-Rolinska J., Chow E. S., Kretzschmar D. and Giebultowicz J. M. (2015). Manipulations of amyloid precursor protein cleavage disrupt the circadian clock in aging Drosophila. *Neurobiol. Dis.* 77, 117-126. 10.1016/j.nbd.2015.02.012 25766673 PMC4402283

[DMM030627C18] Bloch G., Barnes B. M., Gerkema M. P. and Helm B. (2013). Animal activity around the clock with no overt circadian rhythms: patterns, mechanisms and adaptive value. *Proceedings. Biol. Sci.* 280, 20130019 10.1098/rspb.2013.0019 PMC3712434 23825202

[DMM030627C19] Boeve B. F., Silber M. H., Ferman T. J., Lucas J. A. and Parisi J. E. (2001). Association of REM sleep behavior disorder and neurodegenerative disease may reflect an underlying synucleinopathy. *Mov. Disord.* 16, 622-630. 10.1002/mds.1120 11481685

[DMM030627C20] Bonanni E., Maestri M., Tognoni G., Fabbrini M., Nucciarone B., Manca M. L., Gori S., Iudice A. and Murri L. (2005). Daytime sleepiness in mild and moderate Alzheimer's disease and its relationship with cognitive impairment. *J. Sleep Res.* 14, 311-317. 10.1111/j.1365-2869.2005.00462.x 16120107

[DMM030627C21] Boulos Z. and Terman M. (1980). Food availability and daily biological rhythms. *Neurosci. Biobehav. Rev.* 4, 119-131. 10.1016/0149-7634(80)90010-X 6106914

[DMM030627C22] Braak H. and Braak E. (1994). Morphological criteria for the recognition of Alzheimer's disease and the distribution pattern of cortical changes related to this disorder. *Neurobiol. Aging* 15, 355-356. 10.1016/0197-4580(94)90032-9 7936061

[DMM030627C23] Braak E., Braak H. and Mandelkow E. M. (1994). A sequence of cytoskeleton changes related to the formation of neurofibrillary tangles and neuropil threads. *Acta Neuropathol.* 87, 554-567. 10.1007/BF00293315 7522386

[DMM030627C24] Brody D. L., Magnoni S., Schwetye K. E., Spinner M. L., Esparza T. J., Stocchetti N., Zipfel G. J. and Holtzman D. M. (2008). Amyloid-beta dynamics correlate with neurological status in the injured human brain. *Science* 321, 1221-1224. 10.1126/science.1161591 18755980 PMC2577829

[DMM030627C25] Buhr E. D., Yoo S.-H. and Takahashi J. S. (2010). Temperature as a universal resetting cue for mammalian circadian oscillators. *Science* 330, 379-385. 10.1126/science.1195262 20947768 PMC3625727

[DMM030627C26] Bunger M. K., Wilsbacher L. D., Moran S. M., Clendenin C., Radcliffe L. A., Hogenesch J. B., Simon M. C., Takahashi J. S. and Bradfield C. A. (2000). Mop3 is an essential component of the master circadian pacemaker in mammals. *Cell* 103, 1009-1017. 10.1016/S0092-8674(00)00205-1 11163178 PMC3779439

[DMM030627C27] Buysse D. J., Browman K. E., Monk T. H., Reynolds C. F., Fasiczka A. L. and Kupfer D. J. (1992). Napping and 24-hour sleep/wake patterns in healthy elderly and young adults. *J. Am. Geriatr. Soc.* 40, 779-786. 10.1111/j.1532-5415.1992.tb01849.x 1634721

[DMM030627C28] Camargos E. F., Pandolfi M. B., Freitas M. P. D., Quintas J. L., Lima J. de O., Miranda L. C., Pimentel W., Medeiros-Souza P., Lima Jde O., Miranda L. C. et al. (2011). Trazodone for the treatment of sleep disorders in dementia: an open-label, observational and review study. *Arq. Neuropsiquiatr.* 69, 44-49. 10.1590/S0004-282X2011000100010 21359422

[DMM030627C29] Camargos E. F., Oliveira L. F., Boaventura T. D. V. and Quintas J. L. (2012). Mianserin for the treatment of sleep disorders in patients with dementia: a retrospective open-label study. *J. Clin. Psychopharmacol.* 32, 576-578. 10.1097/JCP.0b013e31825ddf3d 22760353

[DMM030627C30] Camargos E. F., Louzada L. L., Quintas J. L., Naves J. O. S., Louzada F. M. and Nóbrega O. T. (2014). Trazodone improves sleep parameters in Alzheimer disease patients: a randomized, double-blind, and placebo-controlled study. *Am. J. Geriatr. Psychiatry* 22, 1565-1574. 10.1016/j.jagp.2013.12.174 24495406

[DMM030627C31] Carneiro B. T. S. and Araujo J. F. (2012). Food entrainment: major and recent findings. *Front. Behav. Neurosci.* 6, 83 10.3389/fnbeh.2012.00083 23205007 PMC3506962

[DMM030627C32] Catrysse L. and van Loo G. (2017). Inflammation and the metabolic syndrome: the tissue-specific functions of NF-κB. *Trends Cell Biol.* 27, 417-429. 10.1016/j.tcb.2017.01.006 28237661

[DMM030627C33] Cermakian N., Lamont E. W., Boudreau P. and Boivin D. B. (2011). Circadian clock gene expression in brain regions of Alzheimer ‘s disease patients and control subjects. *J. Biol. Rhythm.* 26, 160-170. 10.1177/0748730410395732 21454296

[DMM030627C34] Chavan R., Feillet C., Costa S. S. F., Delorme J. E., Okabe T., Ripperger J. A. and Albrecht U. (2016). Liver-derived ketone bodies are necessary for food anticipation. *Nat. Commun.* 7, 10580 10.1038/ncomms10580 26838474 PMC4742855

[DMM030627C35] Chemelli R. M., Willie J. T., Sinton C. M., Elmquist J. K., Scammell T., Lee C., Richardson J. A., Clay Williams S., Xiong Y., Kisanuki Y. et al. (1999). Narcolepsy in orexin knockout mice: Molecular genetics of sleep regulation. *Cell* 98, 437-451. 10.1016/S0092-8674(00)81973-X 10481909

[DMM030627C36] Chen K.-F., Possidente B., Lomas D. A. and Crowther D. C. (2014). The central molecular clock is robust in the face of behavioural arrhythmia in a Drosophila model of Alzheimer's disease. *Dis. Model Mech.* 7, 445-458. 10.1242/dmm.014134 24574361 PMC3974455

[DMM030627C37] Cho K. (2001). Chronic ‘jet lag’ produces temporal lobe atrophy and spatial cognitive deficits. *Nat. Neurosci.* 4, 567-568. 10.1038/88384 11369936

[DMM030627C38] Citron M., Westaway D., Xia W., Carlson G., Diehl T., Levesque G., Johnson-Wood K., Lee M., Seubert P., Davis A. et al. (1997). Mutant presenilins of Alzheimer's disease increase production of 42-residue amyloid beta-protein in both transfected cells and transgenic mice. *Nat. Med.* 3, 67-72. 10.1038/nm0197-67 8986743

[DMM030627C39] Coogan A. N., Schutová B., Husung S., Furczyk K., Baune B. T., Kropp P., Häßler F. and Thome J. (2013). The circadian system in Alzheimer's disease: disturbances, mechanisms, and opportunities. *Biol. Psychiatry* 74, 333-339. 10.1016/j.biopsych.2012.11.021 23273723

[DMM030627C40] Costello R. B., Lentino C. V., Boyd C. C., O'Connell M. L., Crawford C. C., Sprengel M. L. and Deuster P. A. (2014). The effectiveness of melatonin for promoting healthy sleep: a rapid evidence assessment of the literature. *Nutr. J.* 13, 106 10.1186/1475-2891-13-106 25380732 PMC4273450

[DMM030627C41] Cretenet G., Le Clech M. and Gachon F. (2010). Circadian clock-coordinated 12 hr period rhythmic activation of the IRE1?? Pathway controls lipid metabolism in mouse liver. *Cell Metab.* 11, 47-57. 10.1016/j.cmet.2009.11.002 20074527

[DMM030627C42] Crowther D. C., Kinghorn K. J., Miranda E., Page R., Curry J. A., Duthie F. A., Gubb D. C. and Lomas D. A. (2005). Intraneuronal Ab, non-amyloid aggregates and neurodegeneration in a Drosophila model of Alzheimer's disease. *Neuroscience* 132, 123-135. 10.1016/j.neuroscience.2004.12.025 15780472

[DMM030627C43] Cunnane S., Nugent S., Roy M., Courchesne-Loyer A., Croteau E., Tremblay S., Castellano A., Pifferi F., Bocti C., Paquet N. et al. (2011). Brain fuel metabolism, aging, and Alzheimer's disease. *Nutrition* 27, 3-20. 10.1016/j.nut.2010.07.021 21035308 PMC3478067

[DMM030627C44] Cuyvers E. and Sleegers K. (2016). Genetic variations underlying Alzheimer's disease: evidence from genome-wide association studies and beyond. *Lancet Neurol.* 15, 857-868. 10.1016/S1474-4422(16)00127-7 27302364

[DMM030627C45] Damiola F., Le Minh N., Preitner N., Kornmann B., Fleury-Olela F. and Schibler U. (2000). Restricted feeding uncouples circadian oscillators in peripheral tissues from the central pacemaker in the suprachiasmatic nucleus. *Genes Dev.* 14, 2950-2961. 10.1101/gad.183500 11114885 PMC317100

[DMM030627C46] de Lecea L., Kilduff T. S., Peyron C., Gao X., Foye P. E., Danielson P. E., Fukuhara C., Battenberg E. L., Gautvik V. T., Bartlett F. S. et al. (1998). The hypocretins: hypothalamus-specific peptides with neuroexcitatory activity. *Proc. Natl. Acad. Sci. USA* 95, 322-327. 10.1073/pnas.95.1.322 9419374 PMC18213

[DMM030627C47] Delezie J. and Challet E. (2011). Interactions between metabolism and circadian clocks: reciprocal disturbances. *Ann. N. Y. Acad. Sci.* 1243, 30-46. 10.1111/j.1749-6632.2011.06246.x 22211891

[DMM030627C48] Di Meco A., Joshi Y. B. and Praticò D. (2014). Sleep deprivation impairs memory, tau metabolism, and synaptic integrity of a mouse model of Alzheimer's disease with plaques and tangles. *Neurobiol. Aging* 35, 1813-1820. 10.1016/j.neurobiolaging.2014.02.011 24629673

[DMM030627C49] Dibner C., Schibler U. and Albrecht U. (2010). The mammalian circadian timing system: organization and coordination of central and peripheral clocks. *Annu. Rev. Physiol.* 72, 517-549. 10.1146/annurev-physiol-021909-135821 20148687

[DMM030627C50] Dickmeis T. (2009). Glucocorticoids and the circadian clock. *J. Endocrinol.* 200, 3-22. 10.1677/JOE-08-0415 18971218

[DMM030627C51] Dijk D. J., Duffy J. F. and Czeisler C. A. (2001). Age-related increase in awakenings: impaired consolidation of nonREM sleep at all circadian phases. *Sleep* 24, 565-577. 10.1093/sleep/24.5.565 11480654

[DMM030627C52] Dissel S., Angadi V., Kirszenblat L., Suzuki Y., Donlea J., Klose M., Koch Z., English D., Winsky-Sommerer R., Van Swinderen B. et al. (2015). Sleep restores behavioral plasticity to drosophila mutants. *Curr. Biol.* 25, 1270-1281. 10.1016/j.cub.2015.03.027 25913403 PMC4465363

[DMM030627C53] Drummond E. and Wisniewski T. (2017). Alzheimer's disease: experimental models and reality. *Acta Neuropathol.* 133, 155-175. 10.1007/s00401-016-1662-x 28025715 PMC5253109

[DMM030627C54] Duncan M. J., Smith J. T., Franklin K. M., Beckett T. L., Murphy M. P., St Clair D. K., Donohue K. D., Striz M. and O'Hara B. F. (2012). Effects of aging and genotype on circadian rhythms, sleep, and clock gene expression in APPxPS1 knock-in mice, a model for Alzheimer's disease. *Exp. Neurol.* 236, 249-258. 10.1016/j.expneurol.2012.05.011 22634208

[DMM030627C55] Edgar D. M. and Dement W. C. (1991). Regularly scheduled voluntary exercise synchronizes the mouse circadian clock. *Am. J. Physiol.* 261, R928-R933. 1928438 10.1152/ajpregu.1991.261.4.R928

[DMM030627C56] Escobar C., Cailotto C., Angeles-Castellanos M., Delgado R. S. and Buijs R. M. (2009). Peripheral oscillators: the driving force for food-anticipatory activity. *Eur. J. Neurosci.* 30, 1665-1675. 10.1111/j.1460-9568.2009.06972.x 19878276

[DMM030627C57] Evans J. A. and Davidson A. J. (2013). Health consequences of circadian disruption in humans and animal models. *Prog. Mol. Biol. Transl. Sci.* 119, 283-323. 10.1016/B978-0-12-396971-2.00010-5 23899601

[DMM030627C58] Fetveit A., Skjerve A. and Bjorvatn B. (2003). Bright light treatment improves sleep in institutionalised elderly--an open trial. *Int. J. Geriatr. Psychiatry* 18, 520-526. 10.1002/gps.852 12789673

[DMM030627C59] Finelli A., Kelkar A., Song H. J., Yang H. and Konsolaki M. (2004). A model for studying Alzheimer's Abeta42-induced toxicity in Drosophila melanogaster. *Mol. Cell. Neurosci.* 26, 365-375. 10.1016/j.mcn.2004.03.001 15234342

[DMM030627C60] Gamble K. L., Berry R., Frank S. J. and Young M. E. (2014). Circadian clock control of endocrine factors. *Nat. Rev. Endocrinol.* 10, 466-475. 10.1038/nrendo.2014.78 24863387 PMC4304769

[DMM030627C61] Gandhi A. V., Mosser E. A., Oikonomou G. and Prober D. A. (2015). Melatonin is required for the circadian regulation of sleep. *Neuron* 85, 1193-1199. 10.1016/j.neuron.2015.02.016 25754820 PMC4851458

[DMM030627C62] Gil-Lozano M., Mingomataj E. L., Wu W. K., Ridout S. A. and Brubaker P. L. (2014). Circadian secretion of the intestinal hormone GLP-1 by the rodent L cell. *Diabetes* 63, 3674-3685. 10.2337/db13-1501 24789917

[DMM030627C63] Gill S., Le H. D., Melkani G. C. and Panda S. (2015). Time-restricted feeding attenuates age-related cardiac decline in Drosophila. *Science* 347, 1265-1269. 10.1126/science.1256682 25766238 PMC4578815

[DMM030627C64] Glaser F. T. and Stanewsky R. (2007). Synchronization of the drosophila circadian clock by temperature cycles. *Cold Spring Harb. Symp. Quant. Biol.* 72, 233-242. 10.1101/sqb.2007.72.046 18419280

[DMM030627C65] Glenner G. G. and Wong C. W. (1984). Alzheimer's disease: initial report of the purification and characterization of a novel cerebrovascular amyloid protein. *Biochem. Biophys. Res. Commun.* 120, 885-890. 10.1016/S0006-291X(84)80190-4 6375662

[DMM030627C66] Goate A., Chartier-Harlin M. C., Mullan M., Brown J., Crawford F., Fidani L., Giuffra L., Haynes A., Irving N. and James L. (1991). Segregation of a missense mutation in the amyloid precursor protein gene with familial Alzheimer's disease. *Nature* 349, 704-706. 10.1038/349704a0 1671712

[DMM030627C67] Gorman M. R. and Yellon S. (2010). Lifespan daily locomotor activity rhythms in a mouse model of amyloid-induced neuropathology. *Chronobiol. Int.* 27, 1159-1177. 10.3109/07420528.2010.485711 20653448

[DMM030627C68] Guerreiro R., Wojtas A., Bras J., Carrasquillo M., Rogaeva E., Majounie E., Cruchaga C., Sassi C., Kauwe J. S. K., Younkin S. et al. (2013). TREM2 variants in Alzheimer's disease. *N. Engl. J. Med.* 368, 117-127. 10.1056/NEJMoa1211851 23150934 PMC3631573

[DMM030627C69] Halliday M., Radford H., Zents K. A. M., Molloy C., Moreno J. A., Verity N. C., Smith E., Ortori C. A., Barrett D. A., Bushell M. et al. (2017). Repurposed drugs targeting eIF2α-P-mediated translational repression prevent neurodegeneration in mice. *Brain* 140, 1768-1783. 10.1093/brain/awx074 28430857 PMC5445255

[DMM030627C70] Hankins M. W., Peirson S. N. and Foster R. G. (2008). Melanopsin: an exciting photopigment. *Trends Neurosci.* 31, 27-36. 10.1016/j.tins.2007.11.002 18054803

[DMM030627C71] Hara R., Wan K., Wakamatsu H., Aida R., Moriya T., Akiyama M. and Shibata S. (2001). Restricted feeding entrains liver clock without participation of the suprachiasmatic nucleus. *Genes Cells* 6, 269-278. 10.1046/j.1365-2443.2001.00419.x 11260270

[DMM030627C72] Hardin P. E. and Panda S. (2013). Circadian timekeeping and output mechanisms in animals. *Curr. Opin. Neurobiol.* 23, 724-731. 10.1016/j.conb.2013.02.018 23731779 PMC3973145

[DMM030627C73] Hardy J. (1997). Amyloid, the presenilins and Alzheimer's disease. *Trends Neurosci.* 20, 154-159. 10.1016/S0166-2236(96)01030-2 9106355

[DMM030627C74] Harper D. G., Stopa E. G., McKee A. C., Satlin A., Harlan P. C., Goldstein R. and Volicer L. (2001). Differential circadian rhythm disturbances in men with Alzheimer disease and frontotemporal degeneration. *Arch. Gen. Psychiatry* 58, 353-360. 10.1001/archpsyc.58.4.353 11296096

[DMM030627C75] Hastings M. H., Duffield G. E., Smith E. J., Maywood E. S. and Ebling F. J. (1998). Entrainment of the circadian system of mammals by nonphotic cues. *Chronobiol. Int.* 15, 425-445. 10.3109/07420529808998700 9787934

[DMM030627C76] Hatori M., Gronfier C., Van Gelder R. N., Bernstein P. S., Carreras J., Panda S., Marks F., Sliney D., Hunt C. E., Hirota T., et al. (2017). Global rise of potential health hazards caused by blue light-induced circadian disruption in modern aging societies. *NPJ Aging Mech. Dis.* 3, 9 10.1038/s41514-017-0010-2 28649427 PMC5473809

[DMM030627C77] Haus E. and Smolensky M. (2006). Biological clocks and shift work: circadian dysregulation and potential long-term effects. *Cancer Causes Control* 17, 489-500. 10.1007/s10552-005-9015-4 16596302

[DMM030627C78] Holscher C. (2014a). Central effects of GLP-1: new opportunities for treatments of neurodegenerative diseases. *J. Endocrinol.* 221, T31-T41. 10.1530/JOE-13-0221 23999914

[DMM030627C79] Holscher C. (2014b). Drugs developed for treatment of diabetes show protective effects in Alzheimer's and Parkinson's diseases. *Acta Physiol. Sin.* 66, 497-510. 10.13294/j.aps.2014.0059 25331995

[DMM030627C80] Holth J. K., Patel T. K. and Holtzman D. M. (2017). Sleep in Alzheimer's Disease–Beyond Amyloid. *Neurobiol. Sleep Circadian Rhythm.* 2, 4-14. 10.1016/j.nbscr.2016.08.002 PMC5312809 28217760

[DMM030627C81] Hsiao K., Chapman P., Nilsen S., Eckman C., Harigaya Y., Younkin S., Yang F. and Cole G. (1996). Correlative memory deficits, Abeta elevation, and amyloid plaques in transgenic mice. *Science* 274, 99-102. 10.1126/science.274.5284.99 8810256

[DMM030627C82] Huang Y., Potter R., Sigurdson W., Santacruz A., Shih S., Ju Y.-E., Kasten T., Morris J. C., Mintun M., Duntley S. et al. (2012). Effects of age and amyloid deposition on Aβ dynamics in the human central nervous system. *Arch. Neurol.* 69, 51-58. 10.1001/archneurol.2011.235 21911660 PMC3254706

[DMM030627C83] Huang C.-C., Chung C.-M., Leu H.-B., Lin L.-Y., Chiu C.-C., Hsu C.-Y., Chiang C.-H., Huang P.-H., Chen T.-J., Lin S.-J. et al. (2014). Diabetes mellitus and the risk of Alzheimer's disease: a nationwide population-based study. *PLoS ONE* 9, e87095 10.1371/journal.pone.0087095 24489845 PMC3906115

[DMM030627C84] Huang N.-Q., Jin H., Zhou S.-Y., Shi J.-S. and Jin F. (2017). TLR4 is a link between diabetes and Alzheimer's disease. *Behav. Brain Res.* 316, 234-244. 10.1016/j.bbr.2016.08.047 27591966

[DMM030627C85] Hut R. A., Kronfeld-Schor N., van der Vinne V. and De la Iglesia H. (2012). In search of a temporal niche: Environmental factors. In *The Neurobiology of Circadian Timing* (ed. Kalsbeek A., Merrow M., Roenneberg T. and Foster R. G.), pp. 281-304. Amsterdam: Elsevier B.V. 10.1016/B978-0-444-59427-3.00017-4 22877672

[DMM030627C86] Hutton M., Heutink P., Lendon C. L., Rizzu P., Baker M., Froelich S., Houlden H., Pickering-Brown S., Chakraverty S., Isaacs A. et al. (1998). Association of missense and 5’-splice-site mutations in tau with the inherited dementia FTDP-17. *Nature* 393, 702-705. 10.1038/31508 9641683

[DMM030627C87] Iijima K., Liu H. P., Chiang A. S., Hearn S. A., Konsolaki M. and Zhong Y. (2004). Dissecting the pathological effects of human Abeta40 and Abeta42 in Drosophila: a potential model for Alzheimer's disease. *Proc. Natl. Acad. Sci. USA* 101, 6623-6628. 10.1073/pnas.0400895101 15069204 PMC404095

[DMM030627C88] Iliff J. J., Wang M., Liao Y., Plogg B. A., Peng W., Gundersen G. A., Benveniste H., Vates G. E., Deane R., Goldman S. A. et al. (2012). A paravascular pathway facilitates CSF flow through the brain parenchyma and the clearance of interstitial solutes, including amyloid β. *Sci. Transl. Med.* 4, 147ra111 10.1126/scitransmed.3003748 PMC3551275 22896675

[DMM030627C89] Iranzo A., Fernández-Arcos A., Tolosa E., Serradell M., Molinuevo J. L., Valldeoriola F., Gelpi E., Vilaseca I., Sánchez-Valle R., Lladó A. et al. (2014). Neurodegenerative disorder risk in idiopathic REM sleep behavior disorder: Study in 174 patients. *PLoS ONE* 9, e89741 10.1371/journal.pone.0089741 24587002 PMC3935943

[DMM030627C90] Janus C., Pearson J., McLaurin J., Mathews P. M., Jiang Y., Schmidt S. D., Chishti M. A., Horne P., Heslin D., French J. et al. (2000). A beta peptide immunization reduces behavioural impairment and plaques in a model of Alzheimer's disease. *Nature* 408, 979-982. 10.1038/35050110 11140685

[DMM030627C91] Jonsson T., Atwal J. K., Steinberg S., Snaedal J., Jonsson P. V., Bjornsson S., Stefansson H., Sulem P., Gudbjartsson D., Maloney J. et al. (2012). A mutation in APP protects against Alzheimer's disease and age-related cognitive decline. *Nature* 488, 96-99. 10.1038/nature11283 22801501

[DMM030627C92] Jonsson T., Stefansson H., Steinberg S., Jonsdottir I., Jonsson P. V., Snaedal J., Bjornsson S., Huttenlocher J., Levey A. I., Lah J. J. et al. (2013). Variant of TREM2 associated with the risk of Alzheimer's disease. *N. Engl. J. Med.* 368, 107-116. 10.1056/NEJMoa1211103 23150908 PMC3677583

[DMM030627C93] Ju Y.-E. S., Lucey B. P. and Holtzman D. M. (2014). Sleep and Alzheimer disease pathology--a bidirectional relationship. *Nat. Rev. Neurol.* 10, 115-119. 10.1038/nrneurol.2013.269 24366271 PMC3979317

[DMM030627C94] Kales H. C., Kim H. M., Zivin K., Valenstein M., Seyfried L. S., Chiang C., Cunningham F., Schneider L. S. and Blow F. C. (2012). Risk of mortality among individual antipsychotics in patients with dementia. *Am. J. Psychiatry* 169, 71-79. 10.1176/appi.ajp.2011.11030347 22193526 PMC4269551

[DMM030627C95] Kang J.-E., Lim M. M., Bateman R. J., Lee J. J., Smyth L. P., Cirrito J. R., Fujiki N., Nishino S. and Holtzman D. M. (2009). Amyloid-beta dynamics are regulated by orexin and the sleep-wake cycle. *Science* 326, 1005-1007. 10.1126/science.1180962 19779148 PMC2789838

[DMM030627C96] Kecklund G. and Axelsson J. (2016). Health consequences of shift work and insufficient sleep. *BMJ* 355, i5210 10.1136/bmj.i5210 27803010

[DMM030627C97] Kewley R. J., Whitelaw M. L. and Chapman-Smith A. (2004). The mammalian basic helix-loop-helix/PAS family of transcriptional regulators. *Int. J. Biochem. Cell Biol.* 36, 189-204. 10.1016/S1357-2725(03)00211-5 14643885

[DMM030627C98] Khabirova E., Chen K.-F., O'Neill J. S. and Crowther D. C. (2016). Flyglow: Single-fly observations of simultaneous molecular and behavioural circadian oscillations in controls and an Alzheimer's model. *Sci. Rep.* 6, 33759 10.1038/srep33759 27658441 PMC5034315

[DMM030627C99] Kishi T., Matsunaga S. and Iwata N. (2015). Suvorexant for primary insomnia: a systematic review and meta-analysis of randomized placebo-controlled trials. *PLoS ONE* 10, e0136910 10.1371/journal.pone.0136910 26317363 PMC4552781

[DMM030627C100] Kleinridders A. (2016). Deciphering brain insulin receptor and insulin-like growth factor 1 receptor signalling. *J. Neuroendocrinol.* 28 10.1111/jne.12433 PMC5129466 27631195

[DMM030627C101] Knight E. M., Brown T. M., Gümüsgöz S., Smith J. C. M., Waters E. J., Allan S. M. and Lawrence C. B. (2013). Age-related changes in core body temperature and activity in triple-transgenic Alzheimer's disease (3xTgAD) mice. *Dis. Model Mech.* 6, 160-170. 10.1242/dmm.010173 22864021 PMC3529348

[DMM030627C102] Knutsson A. (2003). Health disorders of shift workers. *Occup. Med. (Chic. Ill).* 53, 103-108. 10.1093/occmed/kqg048 12637594

[DMM030627C103] Koh K., Zheng X. and Sehgal A. (2006). JETLAG resets the Drosophila circadian clock by promoting light-induced degradation of TIMELESS. *Science* 312, 1809-1812. 10.1126/science.1124951 16794082 PMC2767177

[DMM030627C104] Kondratov R. V. and Antoch M. P. (2007). The clock proteins, aging, and tumorigenesis. *Cold Spring Harb. Symp. Quant. Biol.* 72, 477-482. 10.1101/sqb.2007.72.050 18419307

[DMM030627C105] Kondratova A. A. and Kondratov R. V. (2012). The circadian clock and pathology of the ageing brain. *Nat. Rev. Neurosci.* 13, 325-335. 10.1038/nrn3208 22395806 PMC3718301

[DMM030627C106] Konopka R. J. and Benzer S. (1971). Clock mutants of Drosophila melanogaster. *Proc. Natl. Acad. Sci. USA* 68, 2112-2116. 10.1073/pnas.68.9.2112 5002428 PMC389363

[DMM030627C107] Kopf D. and Frölich L. (2009). Risk of incident Alzheimer's disease in diabetic patients: a systematic review of prospective trials. *J. Alzheimers Dis.* 16, 677-685. 10.3233/JAD-2009-1011 19387104

[DMM030627C108] Kornmann B., Schaad O., Bujard H., Takahashi J. S. and Schibler U. (2007). System-driven and oscillator-dependent circadian transcription in mice with a conditionally active liver clock. *PLoS Biol.* 5, e34 10.1371/journal.pbio.0050034 17298173 PMC1783671

[DMM030627C109] Koss D. J., Robinson L., Drever B. D., Plucińska K., Stoppelkamp S., Veselcic P., Riedel G. and Platt B. (2016). Mutant Tau knock-in mice display frontotemporal dementia relevant behaviour and histopathology. *Neurobiol. Dis.* 91, 105-123. 10.1016/j.nbd.2016.03.002 26949217

[DMM030627C110] Kott J., Leach G. and Yan L. (2012). Direction-dependent effects of chronic “jet-lag” on hippocampal neurogenesis. *Neurosci. Lett.* 515, 177-180. 10.1016/j.neulet.2012.03.048 22465247

[DMM030627C111] Kress B. T., Iliff J. J., Xia M., Wang M., Wei H. S., Zeppenfeld D., Xie L., Kang H., Xu Q., Liew J. A. et al. (2015). Impairment of paravascular clearance pathways in the aging brain. *Ann. Neurol.* 76, 845-861. 10.1002/ana.24271 PMC4245362 25204284

[DMM030627C112] Kunieda T., Minamino T., Katsuno T., Tateno K., Nishi J., Miyauchi H., Orimo M., Okada S. and Komuro I. (2006). Cellular senescence impairs circadian expression of clock genes in vitro and in vivo. *Circ. Res.* 98, 532-539. 10.1161/01.RES.0000204504.25798.a8 16424366

[DMM030627C113] Lack L., Wright H., Kemp K. and Gibbon S. (2005). The treatment of early-morning awakening insomnia with 2 evenings of bright light. *Sleep* 28, 616-623. 10.1093/sleep/28.5.616 16171276

[DMM030627C114] Lambert J. C., Ibrahim-Verbaas C. A., Harold D., Naj A. C., Sims R., Bellenguez C., DeStafano A. L., Bis J. C., Beecham G. W., Grenier-Boley B. et al. (2013). Meta-analysis of 74,046 individuals identifies 11 new susceptibility loci for Alzheimer's disease. *Nat. Genet.* 45, 1452-1458. 10.1038/ng.2802 24162737 PMC3896259

[DMM030627C115] Laposky A., Easton A., Dugovic C., Walisser J., Bradfield C. and Turek F. (2005). Deletion of the mammalian circadian clock gene BMAL1/Mop3 alters baseline sleep architecture and the response to sleep deprivation. *Sleep* 28, 395-410. 10.1093/sleep/28.4.395 16171284

[DMM030627C116] Levine J. D., Funes P., Dowse H. B. and Hall J. C. (2002a). Signal analysis of behavioral and molecular cycles. *BMC Neurosci.* 3, 1 10.1186/1471-2202-3-1 11825337 PMC65508

[DMM030627C117] Levine J. D., Funes P., Dowse H. B. and Hall J. C. (2002b). Resetting the circadian clock by social experience in Drosophila melanogaster. *Science* 298, 2010-2012. 10.1126/science.1076008 12471264

[DMM030627C118] Lewis J., McGowan E., Rockwood J., Melrose H., Nacharaju P., Van Slegtenhorst M., Gwinn-Hardy K., Paul Murphy M., Baker M., Yu X. et al. (2000). Neurofibrillary tangles, amyotrophy and progressive motor disturbance in mice expressing mutant (P301L) tau protein. *Nat. Genet.* 25, 402-405. 10.1038/78078 10932182

[DMM030627C119] Liguori C., Romigi A., Nuccetelli M., Zannino S., Sancesario G. G. M. G., Martorana A., Albanese M., Mercuri N. B., Izzi F., Bernardini S. et al. (2014). Orexinergic system dysregulation, sleep impairment, and cognitive decline in Alzheimer disease. *JAMA Neurol.* 71, 1498-1505. 10.1001/jamaneurol.2014.2510 25322206

[DMM030627C120] Lockie S. H. (2013). Glucagon-like peptide-1 receptor in the brain: role in neuroendocrine control of energy metabolism and treatment target for obesity. *J. Neuroendocrinol.* 25, 597-604. 10.1111/jne.12039 23590331

[DMM030627C121] Long D. M., Blake M. R., Dutta S., Holbrook S. D., Kotwica-Rolinska J., Kretzschmar D. and Giebultowicz J. M. (2014). Relationships between the circadian system and Alzheimer's disease-like symptoms in Drosophila. *PLoS ONE* 9, e106068 10.1371/journal.pone.0106068 25171136 PMC4149435

[DMM030627C122] Macauley S. L., Stanley M., Caesar E. E., Yamada S. A., Raichle M. E., Perez R., Mahan T. E., Sutphen C. L. and Holtzman D. M. (2015). Hyperglycemia modulates extracellular amyloid-β concentrations and neuronal activity in vivo. *J. Clin. Invest.* 125, 2463-2467. 10.1172/JCI79742 25938784 PMC4497756

[DMM030627C123] Maillo C., Martín J., Sebastián D., Hernández-Alvarez M., García-Rocha M., Reina O., Zorzano A., Fernandez M. and Méndez R. (2017). Circadian- and UPR-dependent control of CPEB4 mediates a translational response to counteract hepatic steatosis under ER stress. *Nat. Cell Biol.* 19, 94-105. 10.1038/ncb3461 28092655

[DMM030627C124] Masters C. L., Simms G., Weinman N. A., Multhaup G., McDonald B. L. and Beyreuther K. (1985a). Amyloid plaque core protein in Alzheimer disease and Down syndrome. *Proc. Natl. Acad. Sci. USA* 82, 4245-4249. 10.1073/pnas.82.12.4245 3159021 PMC397973

[DMM030627C125] Masters C. L., Multhaup G., Simms G., Pottgiesser J., Martins R. N. and Beyreuther K. (1985b). Neuronal origin of a cerebral amyloid: neurofibrillary tangles of Alzheimer's disease contain the same protein as the amyloid of plaque cores and blood vessels. *EMBO J.* 4, 2757-2763. 4065091 10.1002/j.1460-2075.1985.tb04000.x PMC554575

[DMM030627C126] Maywood E. S., Fraenkel E., McAllister C. J., Wood N., Reddy A. B., Hastings M. H. and Morton A. J. (2010). Disruption of peripheral circadian timekeeping in a mouse model of Huntington's disease and its restoration by temporally scheduled feeding. *J. Neurosci.* 30, 10199-10204. 10.1523/JNEUROSCI.1694-10.2010 20668203 PMC6633357

[DMM030627C127] McCleery J., Cohen D. A. and Sharpley A. L. (2016). Pharmacotherapies for sleep disturbances in dementia. *Cochrane Database Syst. Rev.* 11, CD009178 10.1002/14651858.CD009178.pub3 27851868 PMC6464889

[DMM030627C128] Mistlberger R. E. and Skene D. J. (2005). Nonphotic entrainment in humans? *J. Biol. Rhythm.* 20, 339-352. 10.1177/0748730405277982 16077153

[DMM030627C129] Miyazaki T., Hashimoto S., Masubuchi S., Honma S. and Honma K. I. (2001). Phase-advance shifts of human circadian pacemaker are accelerated by daytime physical exercise. *Am. J. Physiol. Regul. Integr. Comp. Physiol.* 281, R197-R205. 11404294 10.1152/ajpregu.2001.281.1.R197

[DMM030627C130] Mohawk J. A., Green C. B. and Takahashi J. S. (2012). Central and peripheral circadian clocks in mammals. *Annu. Rev. Neurosci.* 35, 445-462. 10.1146/annurev-neuro-060909-153128 22483041 PMC3710582

[DMM030627C131] Moloney A. M., Griffin R. J., Timmons S., O'Connor R., Ravid R. and O'Neill C. (2010a). Defects in IGF-1 receptor, insulin receptor and IRS-1/2 in Alzheimer's disease indicate possible resistance to IGF-1 and insulin signalling. *Neurobiol. Aging* 31, 224-243. 10.1016/j.neurobiolaging.2008.04.002 18479783

[DMM030627C132] Moloney A., Sattelle D. B. B., Lomas D. A. A. and Crowther D. C. C. (2010b). Alzheimer's disease: insights from Drosophila melanogaster models. *Trends Biochem. Sci.* 35, 228-235. 10.1016/j.tibs.2009.11.004 20036556 PMC2856915

[DMM030627C133] Münch M. and Bromundt V. (2012). Light and chronobiology: implications for health and disease. *Dialogues Clin. Neurosci.* 14, 448-453. 23393421 10.31887/DCNS.2012.14.4/mmuench PMC3553574

[DMM030627C134] Münch M., Knoblauch V., Blatter K., Schröder C., Schnitzler C., Kräuchi K., Wirz-Justice A. and Cajochen C. (2005). Age-related attenuation of the evening circadian arousal signal in humans. *Neurobiol. Aging* 26, 1307-1319. 10.1016/j.neurobiolaging.2005.03.004 16182904

[DMM030627C135] Musiek E. S. and Holtzman D. M. (2016). Mechanisms linking circadian clocks, sleep, and neurodegeneration. *Science* 354, 1004-1008. 10.1126/science.aah4968 27885006 PMC5219881

[DMM030627C136] Musiek E. S., Lim M. M., Yang G., Bauer A. Q., Qi L., Lee Y., Roh J. H., Ortiz-Gonzalez X., Dearborn J. T., Culver J. P. et al. (2013). Circadian clock proteins regulate neuronal redox homeostasis and neurodegeneration. *J. Clin. Invest.* 123, 5389-5400. 10.1172/JCI70317 24270424 PMC3859381

[DMM030627C137] Musiek E. S., Xiong D. D. and Holtzman D. M. (2015). Sleep, circadian rhythms, and the pathogenesis of Alzheimer disease. *Exp. Mol. Med.* 47, e148 10.1038/emm.2014.121 25766617 PMC4351409

[DMM030627C138] Nakamura T. J., Nakamura W., Yamazaki S., Kudo T., Cutler T., Colwell C. S. and Block G. D. (2011). Age-related decline in circadian output. *J. Neurosci.* 31, 10201-10205. 10.1523/JNEUROSCI.0451-11.2011 21752996 PMC3155746

[DMM030627C139] Nedeltcheva A. V. and Scheer F. A. J. L. (2014). Metabolic effects of sleep disruption, links to obesity and diabetes. *Curr. Opin. Endocrinol. Diabetes. Obes.* 21, 293-298. 10.1097/MED.0000000000000082 24937041 PMC4370346

[DMM030627C140] Newman J. C. and Verdin E. (2014). Ketone bodies as signaling metabolites. *Trends Endocrinol. Metab.* 25, 42-52. 10.1016/j.tem.2013.09.002 24140022 PMC4176946

[DMM030627C141] O'Neill J. S. and Feeney K. A. (2014). Circadian redox and metabolic oscillations in mammalian systems. *Antioxid Redox Signal.* 20, 2966-2981. 10.1089/ars.2013.5582 24063592 PMC4038991

[DMM030627C142] O'Neill J. S. and Reddy A. B. (2012). The essential role of cAMP/Ca2+ signalling in mammalian circadian timekeeping. *Biochem. Soc. Trans.* 40, 44-50. 10.1042/BST20110691 22260664 PMC3399769

[DMM030627C143] Oddo S., Caccamo A., Shepherd J. D., Murphy M. P., Golde T. E., Kayed R., Metherate R., Mattson M. P., Akbari Y. and LaFerla F. M. (2003). Triple-transgenic model of Alzheimer's disease with plaques and tangles: intracellular Abeta and synaptic dysfunction. *Neuron* 39, 409-421. 10.1016/S0896-6273(03)00434-3 12895417

[DMM030627C144] Osorio R. S., Pirraglia E., Agüera-Ortiz L. F., During E. H., Sacks H., Ayappa I., Walsleben J., Mooney A., Hussain A., Glodzik L. et al. (2011). Greater risk of Alzheimer's disease in older adults with insomnia. *J. Am. Geriatr. Soc.* 59, 559-562. 10.1111/j.1532-5415.2010.03288.x 21391952 PMC3378676

[DMM030627C145] Ott S., Dziadulewicz N. and Crowther D. C. (2015). Iron is a specific cofactor for distinct oxidation- and aggregation-dependent Aβ toxicity mechanisms in a Drosophila model. *Dis Model Mech* 8, 657-667. 10.1242/dmm.019042 26035384 PMC4486857

[DMM030627C146] Pallesen S., Nordhus I. H., Skelton S. H., Bjorvatn B. and Skjerve A. (2005). Bright light treatment has limited effect in subjects over 55 years with mild early morning awakening. *Percept. Mot. Skills* 101, 759-770. 10.2466/pms.101.3.759-770 16491678

[DMM030627C147] Pallier P. N., Maywood E. S., Zheng Z., Chesham J. E., Inyushkin A. N., Dyball R., Hastings M. H. and Morton A. J. (2007). Pharmacological imposition of sleep slows cognitive decline and reverses dysregulation of circadian gene expression in a transgenic mouse model of Huntington's disease. *J. Neurosci.* 27, 7869-7878. 10.1523/JNEUROSCI.0649-07.2007 17634381 PMC6672877

[DMM030627C148] Pan A., Schernhammer E. S., Sun Q. and Hu F. B. (2011). Rotating night shift work and risk of type 2 diabetes: two prospective cohort studies in women. *PLoS Med.* 8, e1001141 10.1371/journal.pmed.1001141 22162955 PMC3232220

[DMM030627C149] Pandi-Perumal S. R., Srinivasan V., Spence D. W. and Cardinali D. P. (2007). Role of the melatonin system in the control of sleep: therapeutic implications. *CNS Drugs* 21, 995-1018. 10.2165/00023210-200721120-00004 18020480

[DMM030627C150] Patton D. F. and Mistlberger R. E. (2013). Circadian adaptations to meal timing: neuroendocrine mechanisms. *Front. Neurosci.* 7, 185 10.3389/fnins.2013.00185 24133410 PMC3796263

[DMM030627C151] Peila R., Rodriguez B. L., Launer L. J., Honolulu-Asia Aging Study (2002). Type 2 diabetes, APOE gene, and the risk for dementia and related pathologies: the Honolulu-Asia Aging Study. *Diabetes* 51, 1256-1262. 10.2337/diabetes.51.4.1256 11916953

[DMM030627C152] Peschel N. and Helfrich-Förster C. (2011). Setting the clock--by nature: circadian rhythm in the fruitfly Drosophila melanogaster. *FEBS Lett.* 585, 1435-1442. 10.1016/j.febslet.2011.02.028 21354415

[DMM030627C153] Peschel N., Chen K.-F. F., Szabo G. and Stanewsky R. (2009). Light-Dependent Interactions between the Drosophila Circadian Clock Factors Cryptochrome, Jetlag, and Timeless. *Curr. Biol.* 19, 241-247. 10.1016/j.cub.2008.12.042 19185492

[DMM030627C154] Pintwala S. and Peever J. (2017). Circuit mechanisms of sleepiness and cataplexy in narcolepsy. *Curr. Opin. Neurobiol.* 44, 50-58. 10.1016/j.conb.2017.02.010 28343142

[DMM030627C155] Plautz J. D., Kaneko M., Hall J. C. and Kay S. A. (1997). Independent photoreceptive circadian clocks throughout Drosophila. *Science* 278, 1632-1635. 10.1126/science.278.5343.1632 9374465

[DMM030627C156] Postuma R. B. and Berg D. (2016). Advances in markers of prodromal Parkinson disease. *Nat. Rev. Neurol.* 12, 622-634. 10.1038/nrneurol.2016.152 27786242

[DMM030627C157] Prince M., Comas-Herrera A., Knapp M., Guerchet M. and Karagiannidou M. (2016). *World Alzheimer Report 2016: improving healthcare for people living with dementia: coverage, quality and costs now and in the future*. London: Alzheimer's Disease International.

[DMM030627C158] Rajan A. and Perrimon N. (2012). Drosophila cytokine unpaired 2 regulates physiological homeostasis by remotely controlling insulin secretion. *Cell* 151, 123-137. 10.1016/j.cell.2012.08.019 23021220 PMC3475207

[DMM030627C159] Rakai B. D., Chrusch M. J., Spanswick S. C., Dyck R. H. and Antle M. C. (2014). Survival of adult generated hippocampal neurons is altered in circadian arrhythmic mice. *PLoS ONE* 9, e99527 10.1371/journal.pone.0099527 24941219 PMC4062413

[DMM030627C160] Reddy A. B. and Maywood E. S. (2007). Circadian rhythms: per2bations in the liver clock. *Curr. Biol.* 17, R292-R294. 10.1016/j.cub.2007.02.031 17437708

[DMM030627C161] Reddy A. B., Maywood E. S., Karp N. A., King V. M., Inoue Y., Gonzalez F. J., Lilley K. S., Kyriacou C. P. and Hastings M. H. (2007). Glucocorticoid signaling synchronizes the liver circadian transcriptome. *Hepatology* 45, 1478-1488. 10.1002/hep.21571 17538967

[DMM030627C162] Rennels M. L., Gregory T. F., Blaumanis O. R., Fujimoto K. and Grady P. A. (1985). Evidence for a ‘paravascular’ fluid circulation in the mammalian central nervous system, provided by the rapid distribution of tracer protein throughout the brain from the subarachnoid space. *Brain Res.* 326, 47-63. 10.1016/0006-8993(85)91383-6 3971148

[DMM030627C163] Rieger D., Stanewsky R. and Helfrich-Förster C. (2003). Cryptochrome, compound eyes, Hofbauer-Buchner eyelets, and ocelli play different roles in the entrainment and masking pathway of the locomotor activity rhythm in the fruit fly Drosophila melanogaster. *J. Biol. Rhythm.* 18, 377-391. 10.1177/0748730403256997 14582854

[DMM030627C164] Rivera E. J., Goldin A., Fulmer N., Tavares R., Wands J. R. and de la Monte S. M. (2005). Insulin and insulin-like growth factor expression and function deteriorate with progression of Alzheimer's disease: link to brain reductions in acetylcholine. *J. Alzheimers Dis.* 8, 247-268. 10.3233/JAD-2005-8304 16340083

[DMM030627C165] Roenneberg T. and Merrow M. (2016). The circadian clock and human health. *Curr. Biol.* 26, R432-R443. 10.1016/j.cub.2016.04.011 27218855

[DMM030627C166] Rogaev E. I., Sherrington R., Rogaeva E. A., Levesque G., Ikeda M., Liang Y., Chi H., Lin C., Holman K., Tsuda T. et al. (1995). Familial Alzheimer's disease in kindreds with missense mutations in a gene on chromosome 1 related to the Alzheimer's disease type 3 gene. *Nature* 376, 775-778. 10.1038/376775a0 7651536

[DMM030627C167] Roh J. H., Jiang H., Finn M. B., Stewart F. R., Mahan T. E., Cirrito J. R., Heda A., Snider B. J., Li M., Yanagisawa M. et al. (2014). Potential role of orexin and sleep modulation in the pathogenesis of Alzheimer's disease. *J. Exp. Med.* 211, 2487-2496. 10.1084/jem.20141788 25422493 PMC4267230

[DMM030627C168] Rothman S. M., Herdener N., Frankola K. A., Mughal M. R. and Mattson M. P. (2013). Chronic mild sleep restriction accentuates contextual memory impairments, and accumulations of cortical Aβ and pTau in a mouse model of Alzheimer's disease. *Brain Res.* 1529, 200-208. 10.1016/j.brainres.2013.07.010 23856323 PMC3779872

[DMM030627C169] Satlin A., Volicer L., Stopa E. G. and Harper D. (1995). Circadian locomotor activity and core-body temperature rhythms in Alzheimer's disease. *Neurobiol. Aging* 16, 765-771. 10.1016/0197-4580(95)00059-N 8532109

[DMM030627C170] Scheer F. A. J. L., Hilton M. F., Mantzoros C. S. and Shea S. A. (2009). Adverse metabolic and cardiovascular consequences of circadian misalignment. *Proc. Natl. Acad. Sci. USA* 106, 4453-4458. 10.1073/pnas.0808180106 19255424 PMC2657421

[DMM030627C171] Schmidt C., Peigneux P. and Cajochen C. (2012). Age-related changes in sleep and circadian rhythms: impact on cognitive performance and underlying neuroanatomical networks. *Front Neurol.* 3, 118 10.3389/fneur.2012.00118 22855682 PMC3405459

[DMM030627C172] Schmutz I., Ripperger J. A., Baeriswyl-Aebischer S. and Albrecht U. (2010). The mammalian clock component PERIOD2 coordinates circadian output by interaction with nuclear receptors. *Genes Dev.* 24, 345-357. 10.1101/gad.564110 20159955 PMC2816734

[DMM030627C173] Schubert M., Brazil D. P., Burks D. J., Kushner J. A., Ye J., Flint C. L., Farhang-Fallah J., Dikkes P., Warot X. M., Rio C. et al. (2003). Insulin receptor substrate-2 deficiency impairs brain growth and promotes tau phosphorylation. *J. Neurosci.* 23, 7084-7092. 12904469 10.1523/JNEUROSCI.23-18-07084.2003 PMC6740672

[DMM030627C174] Sharkey K. M., Carskadon M. A., Figueiro M. G., Zhu Y. and Rea M. S. (2011). Effects of an advanced sleep schedule and morning short wavelength light exposure on circadian phase in young adults with late sleep schedules. *Sleep Med.* 12, 685-692. 10.1016/j.sleep.2011.01.016 21704557 PMC3145013

[DMM030627C175] Sherrington R., Rogaev E. I., Liang Y., Rogaeva E. A., Levesque G., Ikeda M., Chi H., Lin C., Li G., Holman K. et al. (1995). Cloning of a gene bearing missense mutations in early-onset familial Alzheimer's disease. *Nature* 375, 754-760. 10.1038/375754a0 7596406

[DMM030627C176] Simoni A., Wolfgang W., Topping M. P., Kavlie R. G., Stanewsky R. and Albert J. T. (2014). A mechanosensory pathway to the Drosophila circadian clock. *Science* 343, 525-528. 10.1126/science.1245710 24482478

[DMM030627C177] Singleton A. and Hardy J. (2016). The evolution of genetics: Alzheimer's and Parkinson's diseases. *Neuron* 90, 1154-1163. 10.1016/j.neuron.2016.05.040 27311081 PMC4936485

[DMM030627C178] Sperling L. S., Mechanick J. I., Neeland I. J., Herrick C. J., Després J. P., Ndumele C. E., Vijayaraghavan K., Handelsman Y., Puckrein G. A., Araneta M. R. G. et al. (2015). The cardiometabolic health alliance working toward a new care model for the metabolic syndrome. *J. Am. Coll. Cardiol.* 66, 1050-1067. 10.1016/j.jacc.2015.06.1328 26314534

[DMM030627C179] Spiegel K., Tasali E., Leproult R. and Van Cauter E. (2009). Effects of poor and short sleep on glucose metabolism and obesity risk. *Nat. Rev. Endocrinol.* 5, 253-261. 10.1038/nrendo.2009.23 19444258 PMC4457292

[DMM030627C180] Spillantini M. G. and Goedert M. (2013). Tau pathology and neurodegeneration. *Lancet Neurol.* 12, 609-622. 10.1016/S1474-4422(13)70090-5 23684085

[DMM030627C181] Spillantini M. G., Murrell J. R., Goedert M., Farlow M. R., Klug A. and Ghetti B. (1998). Mutation in the tau gene in familial multiple system tauopathy with presenile dementia. *Proc. Natl. Acad. Sci. USA* 95, 7737-7741. 10.1073/pnas.95.13.7737 9636220 PMC22742

[DMM030627C182] Steen E., Terry B. M., Rivera E. J., Cannon J. L., Neely T. R., Tavares R., Xu X. J., Wands J. R. and de la Monte S. M. (2005). Impaired insulin and insulin-like growth factor expression and signaling mechanisms in Alzheimer's disease--is this type 3 diabetes? *J. Alzheimers Dis.* 7, 63-80. 10.3233/JAD-2005-7107 15750215

[DMM030627C183] Sterniczuk R., Dyck R. H., LaFerla F. M. and Antle M. C. (2010). Characterization of the 3xTg-AD mouse model of Alzheimer's disease: part 1. Circadian changes. *Brain Res.* 1348, 139-148. 10.1016/j.brainres.2010.05.013 20471965

[DMM030627C184] Stokkan K. A., Yamazaki S., Tei H., Yoshiyuki S., Menaker M., Sakaki Y. and Menaker M. (2001). Entrainment of the Circadian Clock in the Liver by Feeding. *Science* 291, 490-493. 10.1126/science.291.5503.490 11161204

[DMM030627C185] Stopa E. G., Volicer L., Kuo-Leblanc V., Harper D., Lathi D., Tate B. and Satlin A. (1999). Pathologic evaluation of the human suprachiasmatic nucleus in severe dementia. *J. Neuropathol. Exp. Neurol.* 58, 29-39. 10.1097/00005072-199901000-00004 10068311

[DMM030627C186] Swaab D. F., Fisser B., Kamphorst W. and Troost D. (1988). The human suprachiasmatic nucleus; neuropeptide changes in senium and Alzheimer's disease. *Basic Appl. Histochem.* 32, 43-54. 3291850

[DMM030627C187] Tahara Y., Takatsu Y., Shiraishi T., Kikuchi Y., Yamazaki M., Motohashi H., Muto A., Sasaki H., Haraguchi A., Kuriki D. et al. (2017). Age-related circadian disorganization caused by sympathetic dysfunction in peripheral clock regulation. *NPJ Aging Mech. Dis.* 3, 16030 10.1038/npjamd.2016.30 28721279 PMC5515066

[DMM030627C188] Takeda S., Sato N., Uchio-Yamada K., Sawada K., Kunieda T., Takeuchi D., Kurinami H., Shinohara M., Rakugi H. and Morishita R. (2010). Diabetes-accelerated memory dysfunction via cerebrovascular inflammation and Abeta deposition in an Alzheimer mouse model with diabetes. *Proc. Natl. Acad. Sci. USA* 107, 7036-7041. 10.1073/pnas.1000645107 20231468 PMC2872449

[DMM030627C189] Talbot K., Wang H.-Y., Kazi H., Han L.-Y., Bakshi K. P., Stucky A., Fuino R. L., Kawaguchi K. R., Samoyedny A. J., Wilson R. S. et al. (2012). Demonstrated brain insulin resistance in Alzheimer's disease patients is associated with IGF-1 resistance, IRS-1 dysregulation, and cognitive decline. *J. Clin. Invest.* 122, 1316-1338. 10.1172/JCI59903 22476197 PMC3314463

[DMM030627C190] Tarasoff-Conway J. M., Carare R. O., Osorio R. S., Glodzik L., Butler T., Fieremans E., Axel L., Rusinek H., Nicholson C., Zlokovic B. V. et al. (2015). Clearance systems in the brain—implications for Alzheimer disease. *Nat. Rev. Neurol.* 11, 457-470. 10.1038/nrneurol.2015.119 26195256 PMC4694579

[DMM030627C191] Tate B., Aboody-Guterman K. S., Morris A. M., Walcott E. C., Majocha R. E. and Marotta C. A. (1992). Disruption of circadian regulation by brain grafts that overexpress Alzheimer beta/A4 amyloid. *Proc. Natl. Acad. Sci. USA* 89, 7090-7094. 10.1073/pnas.89.15.7090 1496005 PMC49651

[DMM030627C192] Tatebayashi Y., Miyasaka T., Chui D.-H., Akagi T., Mishima K., Iwasaki K., Fujiwara M., Tanemura K., Murayama M., Ishiguro K. et al. (2002). Tau filament formation and associative memory deficit in aged mice expressing mutant (R406W) human tau. *Proc. Natl. Acad. Sci. USA* 99, 13896-13901. 10.1073/pnas.202205599 12368474 PMC129794

[DMM030627C193] Tranah G. J., Blackwell T., Stone K. L., Ancoli-Israel S., Paudel M. L., Ensrud K. E., Cauley J. A., Redline S., Hillier T. A., Cummings S. R. et al. (2011). Circadian activity rhythms and risk of incident dementia and mild cognitive impairment in older women. *Ann. Neurol.* 70, 722-732. 10.1002/ana.22468 22162057 PMC3244839

[DMM030627C194] van Maanen A., Meijer A. M., van der Heijden K. B. and Oort F. J. (2016). The effects of light therapy on sleep problems: a systematic review and meta-analysis. *Sleep Med. Rev.* 29, 52-62. 10.1016/j.smrv.2015.08.009 26606319

[DMM030627C195] Villegas-Llerena C., Phillips A., Garcia-Reitboeck P., Hardy J. and Pocock J. M. (2016). Microglial genes regulating neuroinflammation in the progression of Alzheimer's disease. *Curr. Opin. Neurobiol.* 36, 74-81. 10.1016/j.conb.2015.10.004 26517285

[DMM030627C196] Volicer L., Harper D. G., Manning B. C., Goldstein R. and Satlin A. (2001). Sundowning and circadian rhythms in Alzheimer's disease. *Am. J. Psychiatry* 158, 704-711. 10.1176/appi.ajp.158.5.704 11329390

[DMM030627C197] Wang K.-C., Woung L.-C., Tsai M.-T., Liu C.-C., Su Y.-H. and Li C.-Y. (2012). Risk of Alzheimer's disease in relation to diabetes: a population-based cohort study. *Neuroepidemiology* 38, 237-244. 10.1159/000337428 22572745

[DMM030627C198] Wehr T. A., Aeschbach D. and Duncan J. (2001). Evidence for a biological dawn and dusk in the human circadian timing system. *J. Physiol.* 535, 937-951. 10.1111/j.1469-7793.2001.t01-1-00937.x 11559786 PMC2278827

[DMM030627C199] Wisor J. P., Edgar D. M., Yesavage J., Ryan H. S., McCormick C. M., Lapustea N. and Murphy G. M. (2005). Sleep and circadian abnormalities in a transgenic mouse model of Alzheimer's disease: a role for cholinergic transmission. *Neuroscience* 131, 375-385. 10.1016/j.neuroscience.2004.11.018 15708480

[DMM030627C200] Wu Y.-H. H., Fischer D. F., Kalsbeek A., Garidou-Boof M.-L. L., van der Vliet J., van Heijningen C., Liu R.-Y. Y., Zhou J.-N. N. and Swaab D. F. (2006). Pineal clock gene oscillation is disturbed in Alzheimer's disease, due to functional disconnection from the ‘master clock’. *FASEB J.* 20, 1874-1876. 10.1096/fj.05-4446fje 16818472

[DMM030627C201] Wyse C. A. and Coogan A. N. (2010). Impact of aging on diurnal expression patterns of CLOCK and BMAL1 in the mouse brain. *Brain Res.* 1337, 21-31. 10.1016/j.brainres.2010.03.113 20382135

[DMM030627C202] Xie L., Kang H., Xu Q., Chen M. J., Liao Y., Thiyagarajan M., O'Donnell J., Christensen D. J., Nicholson C., Iliff J. J. et al. (2013). Sleep drives metabolite clearance from the adult brain. *Science* 342, 373-377. 10.1126/science.1241224 24136970 PMC3880190

[DMM030627C203] Xu K., Zheng X. and Sehgal A. (2008). Regulation of feeding and metabolism by neuronal and peripheral clocks in Drosophila. *Cell Metab.* 8, 289-300. 10.1016/j.cmet.2008.09.006 18840359 PMC2703740

[DMM030627C204] Xu J., Wang L.-L., Dammer E. B., Li C.-B., Xu G., Chen S.-D. and Wang G. (2015). Melatonin for sleep disorders and cognition in dementia: a meta-analysis of randomized controlled trials. *Am. J. Alzheimers. Dis. Other Demen.* 30, 439-447. 10.1177/1533317514568005 25614508 PMC10852893

[DMM030627C205] Yaggi H. K., Araujo A. B. and McKinlay J. B. (2006). Sleep duration as a risk factor for the development of type 2 diabetes. *Diabetes Care* 29, 657-661. 10.2337/diacare.29.03.06.dc05-0879 16505522

[DMM030627C206] Yasumoto Y., Hashimoto C., Nakao R., Yamazaki H., Hiroyama H., Nemoto T., Yamamoto S., Sakurai M., Oike H., Wada N. et al. (2016). Short-term feeding at the wrong time is sufficient to desynchronize peripheral clocks and induce obesity with hyperphagia, physical inactivity and metabolic disorders in mice. *Metabolism* 65, 714-727. 10.1016/j.metabol.2016.02.003 27085778

[DMM030627C207] Yoo S.-H., Yamazaki S., Lowrey P. L., Shimomura K., Ko C. H., Buhr E. D., Siepka S. M., Hong H.-K., Oh W. J., Yoo O. J. et al. (2004). PERIOD2::LUCIFERASE real-time reporting of circadian dynamics reveals persistent circadian oscillations in mouse peripheral tissues. *Proc. Natl. Acad. Sci. USA* 101, 5339-5346. 10.1073/pnas.0308709101 14963227 PMC397382

[DMM030627C208] Yoon I.-Y. Y., Kripke D. F., Elliott J. A., Youngstedt S. D., Rex K. M. and Hauger R. L. (2003). Age-related changes of circadian rhythms and sleep-wake cycles. *J. Am. Geriatr. Soc.* 51, 1085-1091. 10.1046/j.1532-5415.2003.51356.x 12890070

[DMM030627C209] Yurgel M. E., Masek P., DiAngelo J. and Keene A. C. (2015). Genetic dissection of sleep-metabolism interactions in the fruit fly. *J. Comp. Physiol. A. Neuroethol. Sens. Neural. Behav. Physiol.* 201, 869-877. 10.1007/s00359-014-0936-9 25236355 PMC4382448

[DMM030627C210] Zeppenfeld D. M., Simon M., Haswell J. D., D'Abreo D., Murchison C., Quinn J. F., Grafe M. R., Woltjer R. L., Kaye J. and Iliff J. J. (2017). Association of perivascular localization of aquaporin-4 with cognition and Alzheimer disease in aging brains. *JAMA Neurol.* 74, 91-99. 10.1001/jamaneurol.2016.4370 27893874

[DMM030627C211] Zhou J. N., Hofman M. A. and Swaab D. F. (1995). VIP neurons in the human SCN in relation to sex, age, and Alzheimer's disease. *Neurobiol. Aging* 16, 571-576. 10.1016/0197-4580(95)00043-E 8544907

[DMM030627C212] Zhou L., Gao Q., Nie M., Gu J.-L., Hao W., Wang L. and Cao J.-M. (2016). Degeneration and energy shortage in the suprachiasmatic nucleus underlies the circadian rhythm disturbance in ApoE(-/-) mice: implications for Alzheimer's disease. *Sci. Rep.* 6, 36335 10.1038/srep36335 27824104 PMC5099891

[DMM030627C213] Zuber A. M., Centeno G., Pradervand S., Nikolaeva S., Maquelin L., Cardinaux L., Bonny O. and Firsov D. (2009). Molecular clock is involved in predictive circadian adjustment of renal function. *Proc. Natl. Acad. Sci. USA* 106, 16523-16528. 10.1073/pnas.0904890106 19805330 PMC2752602

